# Aqua­(μ-cone-26,28-dibut­oxy-25,27-bis­{*N*-[5-(dimethyl­amino)­naphthalene-1-sulfon­yl]carbamoylmeth­oxy}-5,11,17,23-tetra­kis­(1,1-dimethyl­eth­yl)calix[4]arene(2−))disodium acetonitrile tetra­solvate

**DOI:** 10.1107/S1600536812010768

**Published:** 2012-03-21

**Authors:** Pogisego Dinake, Polina E. Prokhorova, Vladimir S. Talanov, Ray J. Butcher, Galina G. Talanova

**Affiliations:** a Department of Chemistry, Howard University, 525 College Street, NW, Washington, DC 2059, USA

## Abstract

The structure of the title complex, [Na_2_(C_80_H_98_N_4_O_10_S_2_)(H_2_O)]·4CH_3_CN, obtained after crystallization from aceto­nitrile, contains two formula units in the asymmetric unit (*Z*′ = 2) and an estimated four mol­ecules of acetonitrile per calixarene moiety. It is unusual for two Na^+^ ions to occupy the lower rims of the cone calix[4]arene, as in this case, with one Na^+^ ion forming two O→ Na^+^ coordinate bonds with the two but­oxy groups and four such bonds with the two N-dansyl carboxamide groups, forming six dative bonds between Na^+^ and O. On the other hand, the other Na^+^ ion forms only five O→Na^+^ coordinate bonds on the far end of the calix[4]arene lower rim, bringing the two dansyl groups in close proximity with each other. There also appears to be an O→Na^+^ coordination coming from a dangling water mol­ecule. The structure contained both resolved and poorly resolved solvent mol­ecules. The latter were treated using the SQUEEZE routine in *PLATON* [Spek (2009 ▶). Acta Cryst. D*65*, 148–155].

## Related literature

For details of the synthesis, see: Talanova *et al.* (1998 ▶). For refinement details concerning the use of SQUEEZE, see: Spek (2009 ▶). For Hg sensing properties, see: Dinake *et al.* (2010 ▶).
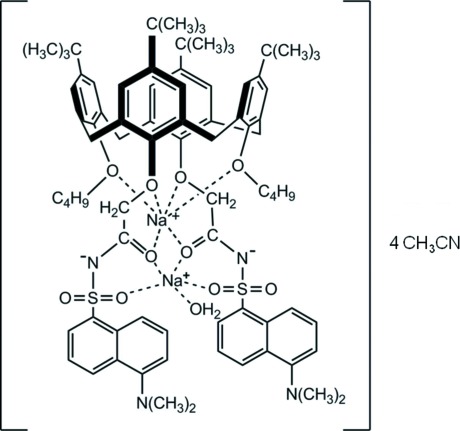


## Experimental

### 

#### Crystal data

[Na_2_(C_80_H_98_N_4_O_10_S_2_)(H_2_O)]·4C_2_H_3_N
*M* *_r_* = 1567.96
Triclinic, 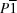

*a* = 18.9264 (3) Å
*b* = 19.3850 (5) Å
*c* = 26.0062 (7) Å
α = 81.799 (2)°
β = 87.108 (2)°
γ = 64.007 (2)°
*V* = 8487.8 (3) Å^3^
*Z* = 4
Cu *K*α radiation
μ = 1.18 mm^−1^
*T* = 123 K
0.82 × 0.25 × 0.07 mm

#### Data collection

Oxford Diffraction Xcalibur Ruby Gemini diffractometer
Absorption correction: analytical (*CrysAlis PRO*; Oxford Diffraction, 2007 ▶) *T*_min_ = 0.527, *T*_max_ = 0.925
65382 measured reflections
34199 independent reflections
26391 reflections with *I* > 2σ(*I*)
*R*_int_ = 0.040

#### Refinement

*R*[*F* ^2^ > 2σ(*F* ^2^)] = 0.080
*wR*(*F* ^2^) = 0.253
*S* = 1.08
34199 reflections
1908 parameters
38 restraints
H-atom parameters constrained
Δρ_max_ = 1.54 e Å^−3^
Δρ_min_ = −0.92 e Å^−3^

### 

Data collection: *CrysAlis PRO* (Oxford Diffraction, 2007 ▶); cell refinement: *CrysAlis PRO*; data reduction: *CrysAlis PRO*; program(s) used to solve structure: *SHELXS97* (Sheldrick, 2008 ▶); program(s) used to refine structure: *SHELXL97* (Sheldrick, 2008 ▶); molecular graphics: *PLATON* (Spek, 2009 ▶); software used to prepare material for publication: *SHELXTL* (Sheldrick, 2008 ▶).

## Supplementary Material

Crystal structure: contains datablock(s) I, global. DOI: 10.1107/S1600536812010768/hg5188sup1.cif

Structure factors: contains datablock(s) I. DOI: 10.1107/S1600536812010768/hg5188Isup2.hkl

Additional supplementary materials:  crystallographic information; 3D view; checkCIF report

## Figures and Tables

**Fig. 1.**
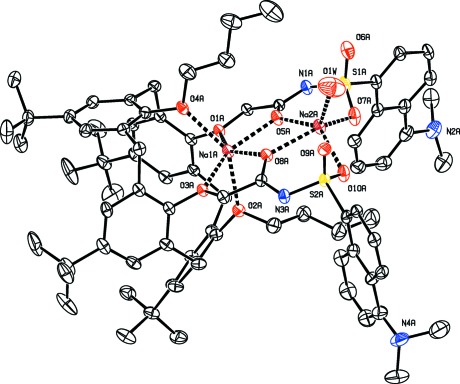
A view of one molecule from the asymmetric unit (Z' = 2) of the title compound, showing the heteroatom numbering. Displacement ellipsoids are drawn at the 30% probability level.

## supplementary crystallographic information

### Comment

The disodium *cone*-5,11,17,23-Tetrakis(1,1-dimethylethyl)-25,-27-bis(*N*-(5-dimethylamminonaphthalene-1-sulfonyl)carbamoylmethoxy)-26,28-dibutoxycalix[4]arene was obtained after crystallization from acetonitrile. The structure contains two formula units in the asymmetric unit (*Z*' = 2) and an estimated four molecules of acetonitrile solvate per calixarene moiety. The title disodium *N*-dansylcarboxamide calix[4]arene complex (Fig. 1) is an unusual crystallographic structure and is the first of such a structure to be reported. It is unusual for two Na^+^ ions to occupy the lower rims of the cone calix[4]arene, as in this case, with one Na^+^ forming two O→Na^+^ coordinate the two butoxy groups and four of such bonds with the two N-dansyl carboxamide groups forming six dative bonds between Na^+^ and O. On the other hand, the other Na^+^ ion forms only five O→Na^+^ coordinate bonds on the far end of the calix[4]arene lower rim, bringing the two dansyl groups in close proximity with each other. There also appears an O→Na^+^ coordination coming from a dangling H_2_O molecule. One Na^+^ ion is in a distorted octahedral coordination geometry, while the other is in a distorted trigonal bipyramidal shape. The two Na^+^ ions are at a bond distance of 3.6001 (12) Å from each other. This compound has been found to have sensorial capabilities towards Hg^2+^ (Dinake *et al.*, 2010) in which the structure of the monosodium form of the title compound was obtained.

The structure contained two resolved acteonitrile solvate molecules and two poorly resolved solvate molecules. The latter were treated using the SQUEEZE routine from *PLATON* (Spek, 2009).

### Experimental

The title compund was synthesized from a *cone* diacid (Talanova *et al.*, 1998) by treatment with (COCl)_2_. The compound was found to possess extreme affinity towards Na^+^ ions and it could therefore not be obtained free from Na^+^ after washing with 0.1 *M* HCl.

### Refinement

H atoms were placed in geometrically idealized positions and constrained to ride on their parent atoms with a C—H distances of 0.95, 0.98 and 0.99%A [*U*ĩso~(H) = 1.2*U*~eq~(CH and CH_2_); *U*ĩso~(H) = 1.5*U*~eq~) for CH_3_]. The water H's were found in the difference Fourier and then constrained with O—H = 0.82 Å and H—O—H angle of 104.5°.

This structure contains *n*-butyl groups. Even though this structure was collected at 123 K the thermal parameters for one of the four *n*-butyl groups were very high. This was attempted to be modeled as disorder but this was not successful. The conclusion is that this group is not well defined and thus was refined isotropically. In addition, the structure contains two resolved acteonitrile molecules and two poorly resolved solvate molecules. The latter were treated using the SQUEEZE routine from *PLATON* (Spek, 2009).

### Crystal data

**Table:**

[Na_2_(C_80_H_98_N_4_O_10_S_2_)(H_2_O)]·4(C_2_H_3_N)	*Z* = 4
*M**_r_* = 1567.96	*F*(000) = 3176
Triclinic, *P*1	*D*_x_ = 1.227 Mg m^−^^3^
Hall symbol: -P 1	Cu *K*α radiation, λ = 1.54178 Å
*a* = 18.9264 (3) Å	Cell parameters from 19166 reflections
*b* = 19.3850 (5) Å	θ = 2.7–75.6°
*c* = 26.0062 (7) Å	µ = 1.18 mm^−^^1^
α = 81.799 (2)°	*T* = 123 K
β = 87.108 (2)°	Plate, colorless
γ = 64.007 (2)°	0.82 × 0.25 × 0.07 mm
*V* = 8487.8 (3) Å^3^	

### Data collection

**Table:**

Oxford Diffraction Xcalibur Ruby Gemini diffractometer	34199 independent reflections
Radiation source: Enhance (Cu) X-ray Source	26391 reflections with *I* > 2σ(*I*)
Graphite monochromator	*R*_int_ = 0.040
Detector resolution: 10.5081 pixels mm^-1^	θ_max_ = 75.7°, θ_min_ = 2.7°
ω scans	*h* = −15→23
Absorption correction: analytical (*CrysAlis PRO*; Oxford Diffraction, 2007)	*k* = −23→24
*T*_min_ = 0.527, *T*_max_ = 0.925	*l* = −31→32
65382 measured reflections	

### Refinement

**Table:**

Refinement on *F*^2^	Primary atom site location: structure-invariant direct methods
Least-squares matrix: full	Secondary atom site location: difference Fourier map
*R*[*F*^2^ > 2σ(*F*^2^)] = 0.080	Hydrogen site location: inferred from neighbouring sites
*wR*(*F*^2^) = 0.253	H-atom parameters constrained
*S* = 1.08	*w* = 1/[σ^2^(*F*_o_^2^) + (0.1615*P*)^2^ + 2.9659*P*] where *P* = (*F*_o_^2^ + 2*F*_c_^2^)/3
34199 reflections	(Δ/σ)_max_ = 0.001
1908 parameters	Δρ_max_ = 1.54 e Å^−^^3^
38 restraints	Δρ_min_ = −0.92 e Å^−^^3^

### Special details

**Table:**

Geometry. All e.s.d.'s (except the e.s.d. in the dihedral angle between two l.s. planes) are estimated using the full covariance matrix. The cell e.s.d.'s are taken into account individually in the estimation of e.s.d.'s in distances, angles and torsion angles; correlations between e.s.d.'s in cell parameters are only used when they are defined by crystal symmetry. An approximate (isotropic) treatment of cell e.s.d.'s is used for estimating e.s.d.'s involving l.s. planes.
Refinement. Refinement of *F*^2^ against ALL reflections. The weighted *R*-factor *wR* and goodness of fit *S* are based on *F*^2^, conventional *R*-factors *R* are based on *F*, with *F* set to zero for negative *F*^2^. The threshold expression of *F*^2^ > σ(*F*^2^) is used only for calculating *R*-factors(gt) *etc*. and is not relevant to the choice of reflections for refinement. *R*-factors based on *F*^2^ are statistically about twice as large as those based on *F*, and *R*- factors based on ALL data will be even larger.

### Fractional atomic coordinates and isotropic or equivalent isotropic displacement parameters (Å^2^)

**Table:**

	*x*	*y*	*z*	*U*_iso_*/*U*_eq_	
S1A	0.51353 (4)	0.61647 (3)	0.36288 (2)	0.03841 (13)	
S2A	0.44161 (4)	0.96872 (3)	0.38756 (2)	0.03441 (12)	
Na1A	0.33272 (5)	0.87110 (5)	0.24731 (4)	0.03182 (19)	
Na2A	0.47429 (3)	0.79912 (5)	0.35234 (4)	0.0384 (2)	
O1A	0.31408 (9)	0.76703 (8)	0.22503 (6)	0.0288 (3)	
O2A	0.21297 (10)	0.90401 (10)	0.29489 (7)	0.0350 (4)	
O3A	0.27596 (9)	1.00639 (8)	0.23513 (6)	0.0285 (3)	
O4A	0.38475 (9)	0.87190 (10)	0.16306 (7)	0.0357 (4)	
O5A	0.41757 (10)	0.75671 (9)	0.29278 (7)	0.0367 (4)	
O6A	0.59331 (12)	0.59701 (13)	0.34715 (9)	0.0534 (5)	
O7A	0.47953 (13)	0.68057 (11)	0.39304 (8)	0.0492 (5)	
O8A	0.39788 (9)	0.91474 (9)	0.29900 (7)	0.0327 (3)	
O9A	0.51710 (11)	0.96324 (12)	0.36985 (7)	0.0452 (4)	
O10A	0.44125 (13)	0.89548 (11)	0.40937 (7)	0.0460 (5)	
O1W	0.59359 (5)	0.80068 (18)	0.34534 (10)	0.1235 (15)	
H1W1	0.6265	0.7559	0.3344	0.185*	
H1W2	0.6147	0.7871	0.3760	0.185*	
N1A	0.45908 (13)	0.62449 (11)	0.31496 (8)	0.0374 (5)	
N2A	0.38431 (17)	0.39210 (16)	0.50674 (10)	0.0546 (6)	
N3A	0.37469 (13)	1.01864 (11)	0.34416 (8)	0.0358 (4)	
N4A	0.23365 (19)	1.1513 (3)	0.57481 (13)	0.0805 (11)	
C1A	0.25576 (13)	0.76883 (12)	0.19343 (9)	0.0286 (4)	
C2A	0.27076 (13)	0.76422 (12)	0.14043 (9)	0.0301 (5)	
C3A	0.21364 (14)	0.76213 (14)	0.10967 (10)	0.0354 (5)	
H3AA	0.2238	0.7571	0.0739	0.042*	
C4A	0.14182 (14)	0.76725 (14)	0.12987 (11)	0.0370 (5)	
C5A	0.12821 (14)	0.77527 (14)	0.18229 (11)	0.0363 (5)	
H5AA	0.0793	0.7800	0.1965	0.044*	
C6A	0.18451 (13)	0.77656 (12)	0.21464 (10)	0.0307 (5)	
C7A	0.08024 (16)	0.76607 (17)	0.09425 (13)	0.0478 (7)	
C8A	0.1171 (2)	0.6949 (2)	0.06615 (18)	0.0767 (12)	
H8AA	0.1613	0.6963	0.0454	0.115*	
H8AB	0.0778	0.6946	0.0433	0.115*	
H8AC	0.1361	0.6481	0.0917	0.115*	
C9A	0.0533 (3)	0.8376 (2)	0.0540 (2)	0.0977 (15)	
H9AA	0.0263	0.8840	0.0713	0.147*	
H9AB	0.0172	0.8356	0.0291	0.147*	
H9AC	0.0990	0.8395	0.0356	0.147*	
C10A	0.0090 (2)	0.7659 (4)	0.1239 (2)	0.0999 (15)	
H10A	−0.0158	0.8123	0.1413	0.150*	
H10B	0.0257	0.7196	0.1499	0.150*	
H10C	−0.0287	0.7655	0.0996	0.150*	
C11A	0.16477 (14)	0.78942 (13)	0.27093 (10)	0.0334 (5)	
H11A	0.1400	0.7561	0.2869	0.040*	
H11B	0.2133	0.7759	0.2909	0.040*	
C12A	0.10853 (14)	0.87415 (14)	0.27209 (10)	0.0335 (5)	
C13A	0.02936 (14)	0.90019 (15)	0.26012 (10)	0.0369 (5)	
H13A	0.0100	0.8631	0.2576	0.044*	
C14A	−0.02267 (14)	0.97858 (16)	0.25176 (11)	0.0394 (6)	
C15A	0.00718 (15)	1.03149 (15)	0.25570 (12)	0.0408 (6)	
H15A	−0.0268	1.0852	0.2487	0.049*	
C16A	0.08550 (14)	1.00849 (14)	0.26958 (11)	0.0372 (5)	
C17A	0.13504 (14)	0.92919 (14)	0.27923 (10)	0.0337 (5)	
C18A	−0.10971 (15)	1.00211 (17)	0.23978 (13)	0.0455 (6)	
C19A	−0.14768 (18)	0.9826 (2)	0.28923 (15)	0.0597 (9)	
H19A	−0.1205	0.9269	0.3011	0.090*	
H19B	−0.2031	0.9971	0.2822	0.090*	
H19C	−0.1439	1.0114	0.3162	0.090*	
C20A	−0.15429 (18)	1.0883 (2)	0.21931 (19)	0.0657 (10)	
H20A	−0.1562	1.1192	0.2465	0.099*	
H20B	−0.2079	1.0997	0.2093	0.099*	
H20C	−0.1273	1.1011	0.1890	0.099*	
C21A	−0.11595 (17)	0.9558 (2)	0.19799 (14)	0.0549 (8)	
H21A	−0.0863	0.9003	0.2095	0.082*	
H21B	−0.0943	0.9699	0.1653	0.082*	
H21C	−0.1713	0.9680	0.1926	0.082*	
C22A	0.11665 (15)	1.06894 (14)	0.27185 (11)	0.0389 (5)	
H22A	0.0756	1.1141	0.2864	0.047*	
H22B	0.1630	1.0463	0.2955	0.047*	
C23A	0.13981 (14)	1.09613 (13)	0.21881 (11)	0.0352 (5)	
C24A	0.08372 (15)	1.15606 (14)	0.18551 (12)	0.0414 (6)	
H24A	0.0308	1.1794	0.1967	0.050*	
C25A	0.10214 (16)	1.18282 (15)	0.13692 (12)	0.0432 (6)	
C26A	0.17925 (15)	1.14529 (14)	0.12037 (11)	0.0393 (5)	
H26A	0.1927	1.1621	0.0868	0.047*	
C27A	0.23755 (13)	1.08366 (13)	0.15146 (10)	0.0330 (5)	
C28A	0.21717 (13)	1.06223 (12)	0.20142 (9)	0.0302 (5)	
C29A	0.04207 (19)	1.25256 (18)	0.10172 (15)	0.0574 (8)	
C30A	−0.0420 (2)	1.2751 (3)	0.1218 (2)	0.0952 (17)	
H30A	−0.0451	1.2875	0.1573	0.143*	
H30B	−0.0554	1.2318	0.1216	0.143*	
H30C	−0.0789	1.3205	0.0993	0.143*	
C31A	0.0618 (2)	1.3207 (2)	0.10166 (19)	0.0735 (11)	
H31A	0.0578	1.3339	0.1370	0.110*	
H31B	0.0247	1.3654	0.0787	0.110*	
H31C	0.1154	1.3067	0.0892	0.110*	
C32A	0.0469 (3)	1.2325 (3)	0.0462 (2)	0.1041 (18)	
H32A	0.0988	1.2231	0.0320	0.156*	
H32B	0.0062	1.2756	0.0243	0.156*	
H32C	0.0387	1.1858	0.0468	0.156*	
C33A	0.31772 (14)	1.03813 (14)	0.12911 (10)	0.0353 (5)	
H33A	0.3570	1.0110	0.1576	0.042*	
H33B	0.3343	1.0738	0.1064	0.042*	
C34A	0.31318 (14)	0.97931 (15)	0.09782 (10)	0.0346 (5)	
C35A	0.27532 (16)	1.00493 (16)	0.04919 (10)	0.0399 (5)	
H35A	0.2581	1.0577	0.0349	0.048*	
C36A	0.26193 (16)	0.95554 (16)	0.02088 (10)	0.0402 (6)	
C37A	0.28557 (15)	0.87945 (15)	0.04342 (10)	0.0386 (5)	
H37A	0.2748	0.8456	0.0255	0.046*	
C38A	0.32436 (13)	0.85071 (14)	0.09118 (9)	0.0330 (5)	
C39A	0.34024 (13)	0.90133 (14)	0.11704 (9)	0.0331 (5)	
C40A	0.22002 (18)	0.98372 (18)	−0.03236 (11)	0.0472 (7)	
C41A	0.1415 (2)	0.9789 (3)	−0.02748 (16)	0.0748 (11)	
H41A	0.1098	1.0100	−0.0009	0.112*	
H41B	0.1510	0.9248	−0.0175	0.112*	
H41C	0.1133	0.9987	−0.0609	0.112*	
C42A	0.2036 (3)	1.0673 (2)	−0.05302 (16)	0.0855 (14)	
H42A	0.1705	1.1017	−0.0285	0.128*	
H42B	0.1764	1.0823	−0.0867	0.128*	
H42C	0.2533	1.0715	−0.0571	0.128*	
C43A	0.2702 (2)	0.9311 (3)	−0.07185 (13)	0.0700 (11)	
H43A	0.2793	0.8776	−0.0597	0.105*	
H43B	0.3208	0.9337	−0.0755	0.105*	
H43C	0.2427	0.9483	−0.1055	0.105*	
C44A	0.34373 (14)	0.76752 (14)	0.11561 (10)	0.0346 (5)	
H44A	0.3629	0.7326	0.0886	0.042*	
H44B	0.3858	0.7499	0.1422	0.042*	
C45A	0.22228 (19)	0.90783 (17)	0.34877 (10)	0.0536 (7)	
H45A	0.1717	0.9448	0.3617	0.064*	
H45B	0.2616	0.9279	0.3520	0.064*	
C46A	0.2480 (2)	0.83112 (18)	0.38188 (10)	0.0653 (10)	
H46A	0.2993	0.7940	0.3699	0.078*	
H46B	0.2092	0.8104	0.3789	0.078*	
C47A	0.2550 (3)	0.8403 (2)	0.43777 (11)	0.0695 (10)	
H47A	0.2894	0.8664	0.4397	0.083*	
H47B	0.2024	0.8741	0.4501	0.083*	
C48A	0.2874 (3)	0.7659 (2)	0.47260 (14)	0.0847 (13)	
H48A	0.2900	0.7755	0.5083	0.127*	
H48B	0.3403	0.7327	0.4613	0.127*	
H48C	0.2533	0.7400	0.4713	0.127*	
C49A	0.46812 (14)	0.8440 (2)	0.14930 (14)	0.0594 (8)	
H49A	0.4806	0.8887	0.1399	0.071*	
H49B	0.4784	0.8160	0.1187	0.071*	
C50A	0.51951 (15)	0.79136 (19)	0.19353 (13)	0.0587 (8)	
H50A	0.5033	0.7496	0.2052	0.070*	
H50B	0.5132	0.8208	0.2229	0.070*	
C51A	0.60476 (16)	0.7561 (2)	0.17791 (16)	0.0754 (11)	
H51A	0.6210	0.7976	0.1655	0.090*	
H51B	0.6118	0.7251	0.1493	0.090*	
C52A	0.6550 (2)	0.7048 (3)	0.2245 (2)	0.114 (2)	
H52A	0.7087	0.6744	0.2132	0.171*	
H52B	0.6329	0.6697	0.2404	0.171*	
H52C	0.6559	0.7371	0.2500	0.171*	
C53A	0.36162 (13)	0.69015 (12)	0.25003 (9)	0.0301 (5)	
H53A	0.3923	0.6567	0.2238	0.036*	
H53B	0.3277	0.6678	0.2682	0.036*	
C54A	0.41714 (13)	0.69385 (13)	0.28890 (9)	0.0285 (4)	
C55A	0.51325 (15)	0.53437 (13)	0.40287 (10)	0.0351 (5)	
C56A	0.58467 (16)	0.47611 (15)	0.41798 (10)	0.0396 (5)	
H56A	0.6313	0.4781	0.4043	0.047*	
C57A	0.58920 (17)	0.41232 (15)	0.45425 (11)	0.0419 (6)	
H57A	0.6391	0.3726	0.4660	0.050*	
C58A	0.52261 (17)	0.40770 (15)	0.47234 (10)	0.0414 (6)	
H58A	0.5266	0.3653	0.4973	0.050*	
C59A	0.44761 (16)	0.46456 (15)	0.45481 (10)	0.0390 (5)	
C60A	0.37674 (19)	0.45871 (17)	0.47294 (11)	0.0467 (6)	
C61A	0.30504 (18)	0.51978 (19)	0.45956 (13)	0.0521 (7)	
H61A	0.2583	0.5168	0.4721	0.063*	
C62A	0.30007 (18)	0.58614 (18)	0.42786 (12)	0.0505 (7)	
H62A	0.2498	0.6278	0.4195	0.061*	
C63A	0.36558 (16)	0.59297 (15)	0.40849 (11)	0.0420 (6)	
H63A	0.3602	0.6387	0.3866	0.050*	
C64A	0.44164 (15)	0.53202 (13)	0.42096 (9)	0.0355 (5)	
C65A	0.4193 (2)	0.3187 (2)	0.48626 (16)	0.0660 (9)	
H65A	0.4564	0.3202	0.4589	0.099*	
H65B	0.3780	0.3094	0.4717	0.099*	
H65C	0.4473	0.2770	0.5143	0.099*	
C66A	0.3173 (2)	0.3993 (3)	0.53831 (16)	0.0740 (10)	
H66A	0.2993	0.4460	0.5553	0.111*	
H66B	0.3322	0.3538	0.5648	0.111*	
H66C	0.2749	0.4031	0.5163	0.111*	
C67A	0.30717 (14)	1.04375 (13)	0.26547 (9)	0.0325 (5)	
H67A	0.2638	1.0831	0.2831	0.039*	
H67B	0.3334	1.0703	0.2423	0.039*	
C68A	0.36576 (13)	0.98418 (13)	0.30566 (9)	0.0287 (4)	
C69A	0.41109 (14)	1.03086 (13)	0.43660 (9)	0.0326 (5)	
C70A	0.45821 (14)	1.06473 (14)	0.44613 (9)	0.0335 (5)	
H70A	0.5050	1.0550	0.4270	0.040*	
C71A	0.43636 (16)	1.11445 (15)	0.48488 (11)	0.0396 (5)	
H71A	0.4681	1.1391	0.4913	0.048*	
C72A	0.36977 (18)	1.12731 (17)	0.51314 (11)	0.0451 (6)	
H72A	0.3562	1.1606	0.5392	0.054*	
C73A	0.32075 (17)	1.09213 (17)	0.50446 (11)	0.0439 (6)	
C74A	0.24974 (19)	1.1046 (2)	0.53411 (14)	0.0609 (9)	
C75A	0.2014 (2)	1.0747 (3)	0.52080 (16)	0.0693 (10)	
H75A	0.1537	1.0852	0.5390	0.083*	
C76A	0.2208 (2)	1.0290 (2)	0.48102 (16)	0.0644 (9)	
H76A	0.1863	1.0083	0.4728	0.077*	
C77A	0.28779 (18)	1.01326 (19)	0.45372 (13)	0.0512 (7)	
H77A	0.2997	0.9813	0.4270	0.061*	
C78A	0.34064 (15)	1.04397 (15)	0.46461 (10)	0.0383 (5)	
C79A	0.2852 (3)	1.1051 (4)	0.6212 (2)	0.0998 (17)	
H79A	0.3385	1.0739	0.6096	0.150*	
H79B	0.2639	1.0710	0.6400	0.150*	
H79C	0.2867	1.1404	0.6441	0.150*	
C80A	0.1528 (3)	1.1838 (4)	0.5922 (2)	0.113 (2)	
H80A	0.1184	1.2204	0.5640	0.170*	
H80B	0.1485	1.2108	0.6222	0.170*	
H80C	0.1368	1.1420	0.6023	0.170*	
S1B	−0.00248 (4)	0.67528 (4)	0.29971 (4)	0.05278 (19)	
S2B	−0.03275 (4)	0.36767 (4)	0.26417 (4)	0.0613 (2)	
Na1B	0.006053 (16)	0.51233 (3)	0.258386 (17)	0.0751 (5)	
Na2B	0.21544 (5)	0.41753 (5)	0.24189 (4)	0.0349 (2)	
O1B	0.26323 (9)	0.51072 (8)	0.23582 (7)	0.0317 (3)	
O2B	0.22183 (10)	0.43315 (10)	0.15011 (8)	0.0408 (4)	
O3B	0.24895 (8)	0.28883 (9)	0.23450 (7)	0.0310 (3)	
O4B	0.28388 (9)	0.37151 (9)	0.32189 (7)	0.0337 (4)	
O5B	0.12010 (10)	0.53136 (10)	0.26675 (9)	0.0471 (5)	
O6B	−0.04104 (12)	0.63448 (14)	0.27932 (12)	0.0635 (7)	
O7B	−0.04735 (13)	0.75726 (13)	0.29601 (11)	0.0615 (6)	
O8B	0.10564 (10)	0.39458 (10)	0.24480 (10)	0.0498 (5)	
O9B	−0.07649 (15)	0.41413 (15)	0.21792 (15)	0.0868 (10)	
O10B	−0.03405 (15)	0.41209 (14)	0.30427 (14)	0.0848 (9)	
O2W	−0.06963 (8)	0.55631 (4)	0.17973 (4)	0.258 (4)*	
H2W1	−0.0793	0.5208	0.1728	0.387*	
H2W2	−0.0845	0.5949	0.1710	0.387*	
N1B	0.08127 (13)	0.66047 (13)	0.27480 (11)	0.0472 (6)	
N2B	0.1262 (3)	0.6200 (3)	0.53855 (18)	0.0932 (12)	
N3B	0.05368 (14)	0.30579 (14)	0.25186 (12)	0.0530 (6)	
N4B	−0.07703 (17)	0.07382 (17)	0.39399 (13)	0.0599 (7)	
C1B	0.33879 (12)	0.50430 (12)	0.22439 (10)	0.0298 (5)	
C2B	0.39294 (13)	0.48242 (12)	0.26478 (10)	0.0306 (5)	
C3B	0.46769 (13)	0.47863 (13)	0.25263 (10)	0.0320 (5)	
H3BA	0.5052	0.4642	0.2799	0.038*	
C4B	0.48837 (13)	0.49545 (13)	0.20195 (10)	0.0335 (5)	
C5B	0.43318 (14)	0.51460 (13)	0.16267 (10)	0.0338 (5)	
H5BA	0.4468	0.5255	0.1278	0.041*	
C6B	0.35810 (13)	0.51839 (12)	0.17270 (10)	0.0313 (5)	
C7B	0.56901 (14)	0.49492 (15)	0.18909 (12)	0.0398 (6)	
C8B	0.60471 (18)	0.4520 (2)	0.14241 (15)	0.0604 (8)	
H8BA	0.6102	0.3989	0.1500	0.091*	
H8BB	0.6565	0.4509	0.1353	0.091*	
H8BC	0.5703	0.4788	0.1119	0.091*	
C9B	0.62623 (18)	0.4576 (3)	0.23493 (16)	0.0680 (10)	
H9BA	0.6046	0.4864	0.2645	0.102*	
H9BB	0.6766	0.4585	0.2252	0.102*	
H9BC	0.6344	0.4039	0.2446	0.102*	
C10B	0.55740 (17)	0.57857 (18)	0.17609 (17)	0.0582 (9)	
H10D	0.5349	0.6062	0.2060	0.087*	
H10E	0.5216	0.6040	0.1461	0.087*	
H10F	0.6082	0.5792	0.1678	0.087*	
C11B	0.30293 (14)	0.53119 (13)	0.12810 (10)	0.0341 (5)	
H11C	0.3054	0.5710	0.1006	0.041*	
H11D	0.2482	0.5501	0.1407	0.041*	
C12B	0.32604 (14)	0.45578 (13)	0.10580 (10)	0.0336 (5)	
C13B	0.38980 (15)	0.43093 (14)	0.07186 (10)	0.0365 (5)	
H13B	0.4144	0.4640	0.0610	0.044*	
C14B	0.41822 (16)	0.35965 (15)	0.05368 (10)	0.0381 (5)	
C15B	0.38314 (16)	0.31082 (14)	0.07236 (10)	0.0370 (5)	
H15B	0.4033	0.2609	0.0616	0.044*	
C16B	0.32016 (14)	0.33238 (14)	0.10590 (10)	0.0349 (5)	
C17B	0.28948 (14)	0.40753 (14)	0.12043 (10)	0.0345 (5)	
C18B	0.48584 (18)	0.33250 (16)	0.01536 (12)	0.0470 (6)	
C19B	0.4575 (3)	0.3132 (3)	−0.03287 (14)	0.0704 (9)	
H19D	0.4408	0.2722	−0.0223	0.106*	
H19E	0.5006	0.2957	−0.0576	0.106*	
H19F	0.4132	0.3596	−0.0493	0.106*	
C20B	0.5538 (2)	0.2603 (2)	0.04142 (17)	0.0678 (10)	
H20D	0.5707	0.2723	0.0727	0.102*	
H20E	0.5978	0.2430	0.0173	0.102*	
H20F	0.5368	0.2192	0.0511	0.102*	
C21B	0.5139 (2)	0.39474 (19)	−0.00341 (14)	0.0578 (8)	
H21D	0.5297	0.4104	0.0266	0.087*	
H21E	0.4711	0.4398	−0.0224	0.087*	
H21F	0.5589	0.3739	−0.0264	0.087*	
C22B	0.28957 (15)	0.27357 (14)	0.12793 (10)	0.0362 (5)	
H22C	0.2352	0.3010	0.1402	0.043*	
H22D	0.2886	0.2437	0.1003	0.043*	
C23B	0.34173 (13)	0.21854 (13)	0.17282 (10)	0.0320 (5)	
C24B	0.41299 (14)	0.15695 (13)	0.16328 (10)	0.0350 (5)	
H24B	0.4265	0.1474	0.1285	0.042*	
C25B	0.46491 (14)	0.10904 (13)	0.20324 (10)	0.0342 (5)	
C26B	0.44493 (13)	0.12565 (13)	0.25397 (10)	0.0322 (5)	
H26B	0.4807	0.0946	0.2815	0.039*	
C27B	0.37391 (13)	0.18665 (12)	0.26553 (9)	0.0291 (4)	
C28B	0.32219 (13)	0.23060 (12)	0.22410 (10)	0.0300 (5)	
C29B	0.54265 (15)	0.03962 (15)	0.19378 (12)	0.0412 (6)	
C30B	0.5440 (2)	−0.03375 (17)	0.22678 (15)	0.0619 (9)	
H30D	0.5459	−0.0295	0.2637	0.093*	
H30E	0.5905	−0.0793	0.2182	0.093*	
H30F	0.4965	−0.0390	0.2194	0.093*	
C31B	0.55011 (19)	0.02481 (18)	0.13690 (13)	0.0541 (8)	
H31D	0.5474	0.0710	0.1143	0.081*	
H31E	0.5071	0.0133	0.1279	0.081*	
H31F	0.6006	−0.0193	0.1322	0.081*	
C32B	0.61116 (19)	0.0550 (2)	0.20686 (19)	0.0720 (11)	
H32D	0.6089	0.1012	0.1845	0.108*	
H32E	0.6607	0.0104	0.2012	0.108*	
H32F	0.6082	0.0635	0.2433	0.108*	
C33B	0.35632 (13)	0.20541 (13)	0.32090 (10)	0.0318 (5)	
H33C	0.3725	0.1569	0.3453	0.038*	
H33D	0.2991	0.2368	0.3246	0.038*	
C34B	0.40062 (13)	0.25007 (13)	0.33382 (9)	0.0312 (5)	
C35B	0.48024 (14)	0.21088 (14)	0.34609 (10)	0.0332 (5)	
H35B	0.5046	0.1559	0.3495	0.040*	
C36B	0.52639 (14)	0.24894 (15)	0.35371 (10)	0.0352 (5)	
C37B	0.48935 (13)	0.32943 (14)	0.34627 (9)	0.0330 (5)	
H37B	0.5196	0.3567	0.3500	0.040*	
C38B	0.40985 (13)	0.37187 (13)	0.33356 (9)	0.0316 (5)	
C39B	0.36485 (13)	0.33107 (13)	0.33017 (9)	0.0293 (4)	
C40B	0.61283 (15)	0.20154 (17)	0.37079 (12)	0.0434 (6)	
C41B	0.65415 (16)	0.2539 (2)	0.37139 (15)	0.0559 (8)	
H41D	0.6262	0.2929	0.3945	0.084*	
H41E	0.6543	0.2796	0.3361	0.084*	
H41F	0.7084	0.2225	0.3839	0.084*	
C42B	0.6157 (2)	0.1605 (3)	0.42614 (15)	0.0680 (10)	
H42D	0.5929	0.1992	0.4501	0.102*	
H42E	0.6704	0.1261	0.4363	0.102*	
H42F	0.5856	0.1301	0.4273	0.102*	
C43B	0.6563 (2)	0.1420 (2)	0.33456 (18)	0.0769 (12)	
H43D	0.6543	0.1684	0.2992	0.115*	
H43E	0.6315	0.1070	0.3349	0.115*	
H43F	0.7112	0.1122	0.3461	0.115*	
C44B	0.37512 (14)	0.45953 (13)	0.32037 (10)	0.0323 (5)	
H44C	0.3175	0.4821	0.3251	0.039*	
H44D	0.3973	0.4806	0.3443	0.039*	
C45B	0.15266 (19)	0.4740 (3)	0.11606 (19)	0.0959 (15)*	
H45C	0.1493	0.4367	0.0951	0.115*	
H45D	0.1605	0.5142	0.0917	0.115*	
C46B	0.0758 (3)	0.5120 (5)	0.14246 (19)	0.151 (3)*	
H46C	0.0785	0.5487	0.1643	0.181*	
H46D	0.0654	0.4723	0.1654	0.181*	
C47B	0.00954 (15)	0.5551 (2)	0.10306 (11)	0.208 (5)*	
H47C	−0.0357	0.5439	0.1137	0.250*	
H47D	−0.0078	0.6115	0.1012	0.250*	
C48B	0.0376 (7)	0.5299 (8)	0.0505 (2)	0.397 (13)*	
H48D	−0.0047	0.5589	0.0247	0.595*	
H48E	0.0527	0.4743	0.0522	0.595*	
H48F	0.0831	0.5400	0.0405	0.595*	
C49B	0.24115 (15)	0.40263 (15)	0.36751 (10)	0.0452 (6)	
H49C	0.1891	0.4449	0.3563	0.054*	
H49D	0.2701	0.4254	0.3844	0.054*	
C50B	0.22906 (16)	0.34466 (18)	0.40667 (13)	0.0564 (8)	
H50C	0.2791	0.2968	0.4114	0.068*	
H50D	0.2165	0.3653	0.4404	0.068*	
C51B	0.1662 (2)	0.3241 (2)	0.39340 (15)	0.0677 (9)	
H51C	0.1812	0.2967	0.3624	0.081*	
H51D	0.1168	0.3717	0.3852	0.081*	
C52B	0.1538 (4)	0.2721 (4)	0.4393 (3)	0.154 (3)	
H52D	0.1037	0.2700	0.4348	0.231*	
H52E	0.1529	0.2932	0.4716	0.231*	
H52F	0.1969	0.2198	0.4411	0.231*	
C53B	0.21057 (14)	0.58709 (13)	0.24726 (12)	0.0405 (6)	
H53C	0.2029	0.6253	0.2159	0.049*	
H53D	0.2337	0.6010	0.2751	0.049*	
C54B	0.13147 (15)	0.58940 (14)	0.26465 (12)	0.0404 (6)	
C55B	0.02160 (19)	0.6349 (2)	0.36650 (16)	0.0594 (8)	
C56B	0.0184 (3)	0.5659 (2)	0.38267 (19)	0.0759 (11)	
H56B	−0.0038	0.5455	0.3605	0.091*	
C57B	0.0485 (3)	0.5245 (3)	0.43286 (19)	0.0816 (12)	
H57B	0.0483	0.4756	0.4435	0.098*	
C58B	0.0771 (3)	0.5550 (3)	0.46467 (19)	0.0771 (11)	
H58B	0.1000	0.5261	0.4971	0.093*	
C59B	0.0738 (2)	0.6301 (2)	0.45087 (18)	0.0672 (10)	
C60B	0.0987 (2)	0.6634 (3)	0.48673 (17)	0.0723 (10)	
C61B	0.1011 (3)	0.7335 (3)	0.4726 (2)	0.0791 (12)	
H61B	0.1170	0.7562	0.4970	0.095*	
C62B	0.0792 (2)	0.7716 (2)	0.42102 (19)	0.0702 (10)	
H62B	0.0831	0.8189	0.4109	0.084*	
C63B	0.05304 (19)	0.7425 (2)	0.38582 (16)	0.0592 (8)	
H63B	0.0377	0.7699	0.3519	0.071*	
C64B	0.04862 (17)	0.67105 (19)	0.39975 (15)	0.0548 (8)	
C65B	0.0655 (5)	0.6177 (5)	0.5711 (3)	0.134 (2)	
H65D	0.0280	0.6096	0.5510	0.201*	
H65E	0.0384	0.6669	0.5853	0.201*	
H65F	0.0876	0.5751	0.5996	0.201*	
C66B	0.1772 (4)	0.6390 (4)	0.5667 (2)	0.110 (2)	
H66D	0.2203	0.6393	0.5443	0.166*	
H66E	0.1988	0.6003	0.5974	0.166*	
H66F	0.1473	0.6903	0.5776	0.166*	
C67B	0.18976 (13)	0.26105 (13)	0.24091 (11)	0.0354 (5)	
H67C	0.2016	0.2221	0.2723	0.043*	
H67D	0.1888	0.2361	0.2104	0.043*	
C68B	0.11111 (15)	0.32850 (15)	0.24627 (11)	0.0394 (6)	
C69B	−0.07567 (17)	0.30435 (17)	0.28910 (16)	0.0560 (8)	
C70B	−0.14417 (17)	0.3182 (2)	0.26614 (18)	0.0644 (10)	
H70B	−0.1677	0.3611	0.2399	0.077*	
C71B	−0.18059 (17)	0.2686 (2)	0.28132 (17)	0.0622 (10)	
H71B	−0.2286	0.2783	0.2653	0.075*	
C72B	−0.14672 (17)	0.20683 (18)	0.31911 (15)	0.0554 (8)	
H72B	−0.1717	0.1739	0.3289	0.067*	
C73B	−0.07589 (17)	0.19070 (18)	0.34392 (13)	0.0502 (7)	
C74B	−0.03831 (18)	0.12351 (19)	0.38177 (14)	0.0529 (7)	
C75B	0.0322 (2)	0.1075 (2)	0.40296 (16)	0.0654 (9)	
H75B	0.0579	0.0616	0.4267	0.078*	
C76B	0.0677 (2)	0.1585 (2)	0.39010 (18)	0.0687 (10)	
H76B	0.1164	0.1470	0.4059	0.082*	
C77B	0.03293 (19)	0.22359 (19)	0.35536 (16)	0.0589 (8)	
H77B	0.0569	0.2579	0.3482	0.071*	
C78B	−0.03867 (16)	0.24120 (17)	0.32965 (14)	0.0515 (7)	
C79B	−0.1335 (2)	0.0987 (3)	0.43592 (17)	0.0743 (11)	
H79D	−0.1642	0.1551	0.4299	0.111*	
H79E	−0.1051	0.0839	0.4692	0.111*	
H79F	−0.1689	0.0739	0.4367	0.111*	
C80B	−0.0254 (2)	−0.0087 (2)	0.40292 (18)	0.0699 (10)	
H80D	0.0144	−0.0221	0.3758	0.105*	
H80E	−0.0563	−0.0379	0.4021	0.105*	
H80F	0.0006	−0.0217	0.4370	0.105*	
N1S	0.6342 (2)	0.2529 (3)	0.1977 (3)	0.136 (2)	
C11S	0.5674 (2)	0.2728 (2)	0.19960 (18)	0.0694 (11)	
C12S	0.48415 (16)	0.29893 (15)	0.20309 (12)	0.0421 (6)	
H12A	0.4687	0.2659	0.1857	0.063*	
H12B	0.4574	0.3525	0.1862	0.063*	
H12C	0.4693	0.2963	0.2397	0.063*	
N2S	−0.02722 (19)	1.0791 (2)	0.09920 (16)	0.0775 (10)	
C21S	0.03342 (18)	1.03317 (18)	0.11284 (14)	0.0522 (7)	
C22S	0.11086 (17)	0.97632 (17)	0.13048 (13)	0.0469 (6)	
H22E	0.1081	0.9534	0.1662	0.070*	
H22F	0.1462	1.0013	0.1294	0.070*	
H22G	0.1309	0.9357	0.1078	0.070*	
N3S	0.2787 (3)	0.6907 (5)	−0.0167 (2)	0.146 (3)	
C31S	0.2538 (2)	0.7199 (4)	−0.0571 (2)	0.0889 (15)	
C32S	0.2221 (2)	0.7589 (3)	−0.10680 (19)	0.0825 (13)	
H32G	0.1732	0.8052	−0.1029	0.124*	
H32H	0.2111	0.7243	−0.1258	0.124*	
H32I	0.2599	0.7741	−0.1261	0.124*	
N4S	0.7989 (9)	0.7774 (11)	0.1604 (5)	0.405 (5)	
C41S	0.8028 (7)	0.7888 (7)	0.2000 (4)	0.178 (3)	
C42S	0.7803 (5)	0.8001 (6)	0.2453 (4)	0.167 (4)	
H42G	0.7535	0.8559	0.2471	0.251*	
H42H	0.8258	0.7762	0.2690	0.251*	
H42I	0.7439	0.7770	0.2554	0.251*	

### Atomic displacement parameters (Å^2^)

**Table:**

	*U*^11^	*U*^22^	*U*^33^	*U*^12^	*U*^13^	*U*^23^
S1A	0.0492 (3)	0.0285 (3)	0.0396 (3)	−0.0185 (2)	−0.0181 (2)	0.0007 (2)
S2A	0.0456 (3)	0.0288 (2)	0.0305 (3)	−0.0157 (2)	−0.0086 (2)	−0.0077 (2)
Na1A	0.0361 (4)	0.0230 (4)	0.0374 (5)	−0.0123 (3)	−0.0105 (4)	−0.0058 (3)
Na2A	0.0460 (5)	0.0278 (4)	0.0439 (5)	−0.0170 (4)	−0.0163 (4)	−0.0035 (4)
O1A	0.0299 (7)	0.0221 (7)	0.0355 (8)	−0.0107 (6)	−0.0099 (6)	−0.0057 (6)
O2A	0.0357 (8)	0.0330 (8)	0.0390 (9)	−0.0157 (6)	−0.0043 (7)	−0.0085 (7)
O3A	0.0310 (7)	0.0218 (7)	0.0350 (8)	−0.0123 (6)	−0.0092 (6)	−0.0053 (6)
O4A	0.0335 (7)	0.0326 (8)	0.0416 (9)	−0.0134 (6)	−0.0124 (7)	−0.0059 (7)
O5A	0.0416 (8)	0.0266 (7)	0.0444 (9)	−0.0161 (6)	−0.0176 (7)	−0.0021 (7)
O6A	0.0529 (10)	0.0558 (11)	0.0558 (12)	−0.0309 (9)	−0.0163 (9)	0.0098 (9)
O7A	0.0788 (12)	0.0315 (8)	0.0434 (10)	−0.0279 (9)	−0.0244 (9)	−0.0022 (7)
O8A	0.0352 (7)	0.0255 (7)	0.0381 (8)	−0.0118 (6)	−0.0089 (7)	−0.0076 (6)
O9A	0.0455 (9)	0.0472 (10)	0.0379 (9)	−0.0119 (8)	−0.0024 (8)	−0.0173 (8)
O10A	0.0695 (12)	0.0311 (8)	0.0384 (9)	−0.0211 (8)	−0.0170 (9)	−0.0040 (7)
O1W	0.0736 (19)	0.132 (3)	0.177 (4)	−0.041 (2)	−0.003 (2)	−0.067 (3)
N1A	0.0470 (11)	0.0245 (9)	0.0388 (11)	−0.0119 (8)	−0.0162 (9)	−0.0048 (8)
N2A	0.0783 (15)	0.0619 (14)	0.0446 (13)	−0.0479 (12)	0.0042 (12)	−0.0147 (11)
N3A	0.0492 (10)	0.0263 (9)	0.0343 (10)	−0.0173 (8)	−0.0113 (9)	−0.0048 (7)
N4A	0.0526 (15)	0.117 (3)	0.0589 (17)	−0.0223 (17)	0.0216 (14)	−0.0301 (18)
C1A	0.0294 (9)	0.0211 (9)	0.0370 (11)	−0.0108 (8)	−0.0077 (8)	−0.0072 (8)
C2A	0.0302 (10)	0.0230 (9)	0.0365 (11)	−0.0090 (8)	−0.0070 (9)	−0.0080 (8)
C3A	0.0372 (11)	0.0326 (11)	0.0372 (12)	−0.0132 (9)	−0.0093 (9)	−0.0104 (9)
C4A	0.0340 (11)	0.0308 (11)	0.0473 (13)	−0.0126 (9)	−0.0142 (10)	−0.0091 (10)
C5A	0.0316 (10)	0.0290 (11)	0.0502 (14)	−0.0139 (9)	−0.0078 (10)	−0.0064 (10)
C6A	0.0305 (10)	0.0205 (9)	0.0399 (12)	−0.0088 (8)	−0.0068 (9)	−0.0058 (8)
C7A	0.0363 (12)	0.0454 (14)	0.0624 (17)	−0.0135 (11)	−0.0193 (12)	−0.0169 (13)
C8A	0.0577 (17)	0.068 (2)	0.106 (3)	−0.0147 (16)	−0.0360 (19)	−0.044 (2)
C9A	0.104 (2)	0.058 (2)	0.130 (4)	−0.032 (2)	−0.090 (3)	0.016 (2)
C10A	0.0674 (19)	0.173 (4)	0.099 (3)	−0.078 (2)	−0.005 (2)	−0.054 (3)
C11A	0.0349 (10)	0.0270 (10)	0.0403 (12)	−0.0151 (9)	−0.0046 (9)	−0.0040 (9)
C12A	0.0336 (10)	0.0300 (11)	0.0367 (12)	−0.0133 (9)	0.0022 (9)	−0.0065 (9)
C13A	0.0347 (11)	0.0364 (12)	0.0439 (13)	−0.0186 (9)	0.0024 (10)	−0.0089 (10)
C14A	0.0318 (11)	0.0409 (13)	0.0468 (14)	−0.0160 (10)	0.0053 (10)	−0.0107 (10)
C15A	0.0346 (11)	0.0305 (11)	0.0540 (15)	−0.0106 (9)	0.0075 (11)	−0.0105 (10)
C16A	0.0355 (11)	0.0337 (11)	0.0451 (13)	−0.0160 (9)	0.0076 (10)	−0.0132 (10)
C17A	0.0345 (10)	0.0326 (11)	0.0372 (12)	−0.0164 (9)	0.0028 (9)	−0.0093 (9)
C18A	0.0294 (11)	0.0452 (14)	0.0593 (17)	−0.0129 (10)	0.0023 (11)	−0.0110 (12)
C19A	0.0361 (13)	0.072 (2)	0.069 (2)	−0.0217 (14)	0.0116 (13)	−0.0133 (17)
C20A	0.0343 (13)	0.0484 (17)	0.105 (3)	−0.0099 (13)	−0.0076 (16)	−0.0070 (18)
C21A	0.0364 (13)	0.0601 (18)	0.0669 (19)	−0.0180 (13)	−0.0068 (13)	−0.0127 (15)
C22A	0.0388 (11)	0.0305 (11)	0.0501 (14)	−0.0151 (9)	0.0066 (10)	−0.0161 (10)
C23A	0.0328 (10)	0.0252 (10)	0.0504 (14)	−0.0127 (8)	−0.0053 (10)	−0.0113 (9)
C24A	0.0314 (11)	0.0272 (11)	0.0641 (17)	−0.0095 (9)	−0.0085 (11)	−0.0098 (11)
C25A	0.0435 (12)	0.0272 (11)	0.0589 (16)	−0.0143 (10)	−0.0213 (12)	−0.0019 (11)
C26A	0.0462 (12)	0.0319 (11)	0.0423 (13)	−0.0190 (10)	−0.0163 (11)	0.0000 (10)
C27A	0.0350 (10)	0.0269 (10)	0.0421 (12)	−0.0166 (8)	−0.0094 (9)	−0.0061 (9)
C28A	0.0325 (10)	0.0218 (9)	0.0381 (11)	−0.0126 (8)	−0.0101 (9)	−0.0038 (8)
C29A	0.0526 (15)	0.0379 (14)	0.073 (2)	−0.0124 (12)	−0.0314 (15)	0.0051 (14)
C30A	0.0469 (18)	0.065 (2)	0.152 (5)	−0.0135 (17)	−0.043 (2)	0.030 (3)
C31A	0.077 (2)	0.0363 (15)	0.096 (3)	−0.0170 (15)	−0.039 (2)	0.0136 (16)
C32A	0.115 (3)	0.075 (3)	0.094 (3)	−0.011 (3)	−0.074 (3)	0.000 (2)
C33A	0.0384 (11)	0.0347 (11)	0.0385 (12)	−0.0210 (9)	−0.0044 (9)	−0.0041 (9)
C34A	0.0341 (10)	0.0368 (11)	0.0356 (12)	−0.0173 (9)	0.0012 (9)	−0.0075 (9)
C35A	0.0450 (12)	0.0390 (12)	0.0368 (12)	−0.0190 (10)	−0.0012 (10)	−0.0057 (10)
C36A	0.0445 (12)	0.0429 (13)	0.0323 (12)	−0.0172 (11)	−0.0032 (10)	−0.0076 (10)
C37A	0.0422 (12)	0.0398 (12)	0.0348 (12)	−0.0163 (10)	−0.0023 (10)	−0.0127 (10)
C38A	0.0313 (10)	0.0348 (11)	0.0324 (11)	−0.0124 (9)	0.0001 (9)	−0.0094 (9)
C39A	0.0313 (10)	0.0350 (11)	0.0336 (11)	−0.0137 (9)	−0.0023 (9)	−0.0078 (9)
C40A	0.0579 (15)	0.0472 (15)	0.0323 (12)	−0.0181 (13)	−0.0102 (11)	−0.0044 (11)
C41A	0.0589 (19)	0.095 (3)	0.058 (2)	−0.023 (2)	−0.0217 (16)	0.0012 (19)
C42A	0.139 (4)	0.061 (2)	0.054 (2)	−0.041 (2)	−0.040 (2)	0.0069 (16)
C43A	0.077 (2)	0.077 (2)	0.0371 (15)	−0.0145 (19)	−0.0082 (15)	−0.0111 (15)
C44A	0.0341 (11)	0.0312 (11)	0.0383 (12)	−0.0112 (9)	−0.0021 (9)	−0.0137 (9)
C45A	0.0553 (15)	0.0601 (17)	0.0498 (16)	−0.0253 (14)	−0.0043 (13)	−0.0188 (13)
C46A	0.074 (2)	0.0555 (19)	0.0480 (17)	−0.0090 (17)	−0.0086 (16)	−0.0131 (14)
C47A	0.088 (2)	0.072 (2)	0.0480 (18)	−0.033 (2)	−0.0028 (17)	−0.0112 (16)
C48A	0.109 (3)	0.070 (2)	0.065 (2)	−0.027 (2)	−0.009 (2)	−0.017 (2)
C49A	0.0414 (14)	0.0609 (18)	0.077 (2)	−0.0244 (13)	−0.0053 (15)	−0.0024 (16)
C50A	0.0503 (15)	0.0538 (17)	0.073 (2)	−0.0231 (14)	−0.0055 (15)	−0.0077 (15)
C51A	0.0450 (16)	0.073 (2)	0.109 (3)	−0.0229 (16)	0.0008 (19)	−0.025 (2)
C52A	0.065 (3)	0.106 (4)	0.163 (6)	−0.031 (3)	−0.022 (3)	−0.004 (4)
C53A	0.0316 (10)	0.0224 (9)	0.0359 (11)	−0.0098 (8)	−0.0074 (9)	−0.0067 (8)
C54A	0.0304 (9)	0.0265 (10)	0.0294 (10)	−0.0120 (8)	−0.0036 (8)	−0.0060 (8)
C55A	0.0480 (12)	0.0244 (10)	0.0336 (11)	−0.0147 (9)	−0.0122 (10)	−0.0050 (8)
C56A	0.0449 (12)	0.0352 (12)	0.0388 (12)	−0.0161 (10)	−0.0109 (10)	−0.0061 (10)
C57A	0.0503 (13)	0.0272 (11)	0.0435 (13)	−0.0119 (10)	−0.0200 (11)	−0.0006 (10)
C58A	0.0608 (15)	0.0319 (11)	0.0358 (12)	−0.0231 (11)	−0.0097 (11)	−0.0047 (9)
C59A	0.0544 (13)	0.0351 (11)	0.0344 (12)	−0.0227 (10)	−0.0063 (10)	−0.0124 (9)
C60A	0.0678 (16)	0.0499 (14)	0.0378 (13)	−0.0363 (13)	0.0029 (12)	−0.0179 (11)
C61A	0.0521 (14)	0.0599 (16)	0.0538 (16)	−0.0279 (13)	0.0055 (13)	−0.0267 (13)
C62A	0.0480 (14)	0.0500 (15)	0.0519 (16)	−0.0147 (12)	−0.0024 (12)	−0.0231 (13)
C63A	0.0492 (13)	0.0335 (12)	0.0427 (13)	−0.0134 (10)	−0.0058 (11)	−0.0167 (10)
C64A	0.0505 (12)	0.0277 (10)	0.0317 (11)	−0.0168 (9)	−0.0078 (10)	−0.0123 (9)
C65A	0.091 (2)	0.0520 (17)	0.066 (2)	−0.0413 (17)	−0.0145 (18)	−0.0036 (15)
C66A	0.085 (2)	0.102 (3)	0.061 (2)	−0.063 (2)	0.0051 (18)	−0.0156 (19)
C67A	0.0385 (11)	0.0245 (10)	0.0384 (12)	−0.0151 (9)	−0.0097 (9)	−0.0081 (9)
C68A	0.0348 (10)	0.0278 (10)	0.0297 (10)	−0.0187 (8)	−0.0021 (8)	−0.0052 (8)
C69A	0.0411 (11)	0.0295 (10)	0.0294 (10)	−0.0166 (9)	−0.0062 (9)	−0.0046 (8)
C70A	0.0374 (11)	0.0315 (11)	0.0334 (11)	−0.0156 (9)	−0.0042 (9)	−0.0072 (9)
C71A	0.0489 (13)	0.0343 (11)	0.0418 (13)	−0.0217 (10)	−0.0085 (11)	−0.0094 (10)
C72A	0.0560 (15)	0.0404 (13)	0.0363 (13)	−0.0164 (12)	0.0003 (11)	−0.0125 (10)
C73A	0.0449 (13)	0.0457 (14)	0.0379 (13)	−0.0173 (11)	0.0066 (11)	−0.0066 (11)
C74A	0.0420 (15)	0.080 (2)	0.0513 (17)	−0.0184 (15)	0.0077 (13)	−0.0114 (16)
C75A	0.0463 (15)	0.089 (3)	0.072 (2)	−0.0351 (17)	0.0068 (15)	0.0069 (19)
C76A	0.0538 (15)	0.073 (2)	0.076 (2)	−0.0411 (15)	−0.0047 (16)	0.0048 (18)
C77A	0.0527 (14)	0.0532 (15)	0.0557 (17)	−0.0330 (13)	−0.0044 (13)	0.0032 (13)
C78A	0.0412 (12)	0.0378 (12)	0.0368 (12)	−0.0187 (10)	−0.0008 (10)	−0.0026 (10)
C79A	0.078 (3)	0.146 (5)	0.079 (3)	−0.045 (3)	0.016 (2)	−0.042 (3)
C80A	0.072 (3)	0.128 (5)	0.113 (4)	−0.016 (3)	0.045 (3)	−0.038 (3)
S1B	0.0368 (3)	0.0371 (3)	0.0843 (5)	−0.0136 (3)	0.0180 (3)	−0.0222 (3)
S2B	0.0422 (3)	0.0352 (3)	0.1073 (7)	−0.0177 (3)	0.0296 (4)	−0.0195 (4)
Na1B	0.0398 (6)	0.0378 (6)	0.1526 (15)	−0.0173 (5)	0.0169 (7)	−0.0318 (7)
Na2B	0.0294 (4)	0.0241 (4)	0.0554 (6)	−0.0128 (3)	0.0024 (4)	−0.0150 (4)
O1B	0.0272 (7)	0.0210 (7)	0.0495 (9)	−0.0105 (6)	0.0042 (6)	−0.0142 (6)
O2B	0.0323 (8)	0.0347 (8)	0.0584 (11)	−0.0148 (7)	0.0023 (8)	−0.0152 (8)
O3B	0.0272 (7)	0.0224 (7)	0.0473 (9)	−0.0120 (6)	0.0017 (6)	−0.0132 (6)
O4B	0.0295 (7)	0.0300 (7)	0.0447 (9)	−0.0131 (6)	0.0030 (7)	−0.0153 (7)
O5B	0.0296 (8)	0.0304 (8)	0.0857 (14)	−0.0137 (7)	0.0103 (9)	−0.0226 (9)
O6B	0.0361 (9)	0.0506 (12)	0.1038 (19)	−0.0151 (9)	0.0095 (11)	−0.0269 (12)
O7B	0.0470 (11)	0.0440 (11)	0.0907 (17)	−0.0136 (9)	0.0123 (11)	−0.0253 (11)
O8B	0.0328 (8)	0.0284 (8)	0.0918 (16)	−0.0145 (7)	0.0070 (9)	−0.0169 (9)
O9B	0.0567 (13)	0.0455 (13)	0.141 (3)	−0.0166 (11)	0.0258 (16)	0.0140 (15)
O10B	0.0669 (13)	0.0534 (12)	0.149 (3)	−0.0347 (11)	0.0592 (15)	−0.0528 (14)
N1B	0.0401 (11)	0.0301 (10)	0.0738 (16)	−0.0153 (9)	0.0169 (11)	−0.0195 (10)
N2B	0.090 (2)	0.104 (3)	0.090 (3)	−0.046 (2)	0.020 (2)	−0.023 (2)
N3B	0.0385 (10)	0.0400 (11)	0.0897 (19)	−0.0221 (9)	0.0245 (12)	−0.0281 (12)
N4B	0.0565 (14)	0.0558 (15)	0.0691 (18)	−0.0287 (12)	0.0110 (13)	−0.0026 (13)
C1B	0.0281 (9)	0.0205 (9)	0.0450 (12)	−0.0122 (8)	0.0052 (9)	−0.0133 (8)
C2B	0.0324 (10)	0.0216 (9)	0.0428 (12)	−0.0137 (8)	0.0059 (9)	−0.0155 (8)
C3B	0.0317 (10)	0.0243 (9)	0.0437 (12)	−0.0132 (8)	0.0000 (9)	−0.0118 (9)
C4B	0.0288 (10)	0.0247 (10)	0.0487 (13)	−0.0119 (8)	0.0063 (9)	−0.0117 (9)
C5B	0.0335 (10)	0.0238 (10)	0.0444 (13)	−0.0124 (8)	0.0044 (9)	−0.0077 (9)
C6B	0.0294 (10)	0.0196 (9)	0.0457 (13)	−0.0098 (8)	0.0001 (9)	−0.0094 (8)
C7B	0.0288 (10)	0.0372 (12)	0.0545 (15)	−0.0149 (9)	0.0072 (10)	−0.0101 (11)
C8B	0.0418 (14)	0.0656 (19)	0.077 (2)	−0.0218 (13)	0.0224 (14)	−0.0310 (16)
C9B	0.0327 (13)	0.091 (3)	0.072 (2)	−0.0254 (15)	−0.0026 (14)	0.0103 (19)
C10B	0.0419 (13)	0.0458 (15)	0.094 (3)	−0.0271 (12)	0.0138 (15)	−0.0097 (15)
C11B	0.0331 (10)	0.0227 (10)	0.0454 (13)	−0.0106 (8)	−0.0016 (10)	−0.0053 (9)
C12B	0.0348 (11)	0.0275 (10)	0.0369 (12)	−0.0113 (9)	−0.0065 (9)	−0.0046 (9)
C13B	0.0427 (12)	0.0319 (11)	0.0376 (12)	−0.0188 (10)	−0.0009 (10)	−0.0040 (9)
C14B	0.0458 (12)	0.0341 (12)	0.0343 (12)	−0.0168 (10)	0.0007 (10)	−0.0063 (9)
C15B	0.0484 (13)	0.0290 (11)	0.0342 (12)	−0.0159 (10)	−0.0019 (10)	−0.0079 (9)
C16B	0.0393 (11)	0.0327 (11)	0.0363 (12)	−0.0175 (9)	−0.0037 (9)	−0.0081 (9)
C17B	0.0349 (11)	0.0307 (11)	0.0389 (12)	−0.0137 (9)	−0.0036 (9)	−0.0077 (9)
C18B	0.0593 (15)	0.0387 (13)	0.0471 (15)	−0.0248 (12)	0.0145 (12)	−0.0121 (11)
C19B	0.093 (2)	0.091 (2)	0.0494 (17)	−0.055 (2)	0.0292 (17)	−0.0350 (16)
C20B	0.0582 (18)	0.0496 (18)	0.076 (2)	−0.0108 (15)	0.0224 (17)	0.0014 (16)
C21B	0.0681 (18)	0.0467 (15)	0.0629 (19)	−0.0297 (14)	0.0223 (15)	−0.0130 (14)
C22B	0.0420 (11)	0.0327 (11)	0.0408 (12)	−0.0206 (9)	−0.0023 (10)	−0.0106 (9)
C23B	0.0370 (10)	0.0254 (9)	0.0419 (12)	−0.0194 (8)	0.0020 (9)	−0.0109 (9)
C24B	0.0409 (11)	0.0277 (10)	0.0427 (12)	−0.0186 (9)	0.0079 (10)	−0.0144 (9)
C25B	0.0335 (10)	0.0256 (10)	0.0480 (13)	−0.0152 (8)	0.0076 (10)	−0.0135 (9)
C26B	0.0317 (10)	0.0232 (9)	0.0441 (13)	−0.0128 (8)	0.0003 (9)	−0.0088 (9)
C27B	0.0312 (9)	0.0211 (9)	0.0406 (12)	−0.0150 (8)	0.0020 (9)	−0.0104 (8)
C28B	0.0294 (9)	0.0206 (9)	0.0443 (12)	−0.0124 (8)	0.0023 (9)	−0.0128 (8)
C29B	0.0373 (12)	0.0294 (11)	0.0544 (15)	−0.0106 (10)	0.0094 (11)	−0.0148 (10)
C30B	0.0653 (19)	0.0308 (14)	0.068 (2)	−0.0019 (13)	0.0200 (16)	−0.0096 (13)
C31B	0.0551 (16)	0.0403 (14)	0.0618 (18)	−0.0140 (12)	0.0181 (14)	−0.0205 (13)
C32B	0.0363 (14)	0.074 (2)	0.106 (3)	−0.0149 (15)	0.0101 (16)	−0.047 (2)
C33B	0.0333 (10)	0.0245 (9)	0.0410 (12)	−0.0142 (8)	0.0020 (9)	−0.0098 (9)
C34B	0.0356 (10)	0.0297 (10)	0.0326 (11)	−0.0166 (9)	0.0012 (9)	−0.0099 (8)
C35B	0.0350 (11)	0.0298 (10)	0.0357 (11)	−0.0132 (9)	0.0009 (9)	−0.0099 (9)
C36B	0.0317 (10)	0.0414 (12)	0.0339 (11)	−0.0154 (9)	0.0011 (9)	−0.0118 (9)
C37B	0.0342 (10)	0.0399 (11)	0.0335 (11)	−0.0216 (9)	0.0042 (9)	−0.0148 (9)
C38B	0.0345 (10)	0.0332 (10)	0.0330 (11)	−0.0172 (9)	0.0049 (9)	−0.0160 (9)
C39B	0.0286 (9)	0.0271 (10)	0.0352 (11)	−0.0128 (8)	0.0026 (8)	−0.0124 (8)
C40B	0.0315 (11)	0.0484 (14)	0.0506 (15)	−0.0148 (10)	−0.0008 (10)	−0.0159 (12)
C41B	0.0320 (12)	0.0623 (18)	0.076 (2)	−0.0209 (12)	−0.0006 (13)	−0.0164 (16)
C42B	0.0459 (15)	0.085 (2)	0.066 (2)	−0.0271 (16)	−0.0173 (15)	0.0144 (18)
C43B	0.0398 (15)	0.080 (2)	0.098 (3)	−0.0018 (16)	−0.0081 (17)	−0.052 (2)
C44B	0.0353 (10)	0.0297 (10)	0.0388 (12)	−0.0173 (8)	0.0053 (9)	−0.0167 (9)
C49B	0.0405 (12)	0.0426 (13)	0.0535 (15)	−0.0163 (11)	0.0130 (11)	−0.0201 (12)
C50B	0.0497 (15)	0.0583 (18)	0.0561 (18)	−0.0206 (14)	0.0087 (14)	−0.0041 (14)
C51B	0.0688 (19)	0.068 (2)	0.078 (2)	−0.0401 (17)	0.0066 (18)	−0.0130 (18)
C52B	0.154 (4)	0.167 (5)	0.179 (7)	−0.127 (4)	−0.051 (5)	0.070 (5)
C53B	0.0350 (11)	0.0239 (10)	0.0647 (16)	−0.0121 (9)	0.0123 (11)	−0.0188 (10)
C54B	0.0354 (11)	0.0279 (11)	0.0591 (16)	−0.0133 (9)	0.0078 (11)	−0.0141 (10)
C55B	0.0498 (15)	0.0488 (16)	0.083 (2)	−0.0235 (13)	0.0212 (15)	−0.0191 (15)
C56B	0.092 (2)	0.073 (2)	0.085 (3)	−0.055 (2)	0.017 (2)	−0.0202 (19)
C57B	0.104 (3)	0.068 (2)	0.088 (3)	−0.051 (2)	0.023 (2)	−0.018 (2)
C58B	0.081 (2)	0.072 (2)	0.083 (3)	−0.037 (2)	0.012 (2)	−0.014 (2)
C59B	0.0595 (18)	0.0594 (19)	0.087 (3)	−0.0293 (15)	0.0233 (18)	−0.0195 (18)
C60B	0.070 (2)	0.075 (2)	0.071 (2)	−0.0284 (19)	0.0264 (18)	−0.0295 (19)
C61B	0.075 (2)	0.076 (2)	0.095 (3)	−0.034 (2)	0.014 (2)	−0.040 (2)
C62B	0.0608 (18)	0.060 (2)	0.097 (3)	−0.0292 (16)	0.0122 (19)	−0.0266 (19)
C63B	0.0485 (15)	0.0512 (16)	0.082 (2)	−0.0241 (13)	0.0163 (15)	−0.0173 (16)
C64B	0.0407 (13)	0.0530 (16)	0.076 (2)	−0.0213 (12)	0.0236 (13)	−0.0304 (15)
C65B	0.159 (6)	0.153 (6)	0.101 (4)	−0.075 (5)	0.050 (4)	−0.051 (4)
C66B	0.137 (5)	0.107 (4)	0.081 (3)	−0.041 (4)	−0.019 (3)	−0.029 (3)
C67B	0.0346 (10)	0.0297 (10)	0.0499 (14)	−0.0187 (9)	0.0046 (10)	−0.0153 (9)
C68B	0.0361 (11)	0.0331 (11)	0.0558 (15)	−0.0193 (9)	0.0101 (10)	−0.0164 (10)
C69B	0.0391 (13)	0.0374 (13)	0.093 (2)	−0.0186 (11)	0.0265 (14)	−0.0172 (14)
C70B	0.0339 (13)	0.0457 (16)	0.102 (3)	−0.0117 (12)	0.0169 (16)	−0.0002 (17)
C71B	0.0303 (12)	0.0533 (17)	0.098 (3)	−0.0165 (12)	0.0063 (15)	−0.0021 (17)
C72B	0.0373 (13)	0.0461 (15)	0.085 (2)	−0.0208 (11)	0.0170 (14)	−0.0114 (15)
C73B	0.0405 (13)	0.0454 (14)	0.0657 (18)	−0.0187 (11)	0.0193 (13)	−0.0175 (13)
C74B	0.0467 (14)	0.0497 (16)	0.0627 (18)	−0.0208 (12)	0.0129 (13)	−0.0137 (14)
C75B	0.0627 (19)	0.063 (2)	0.072 (2)	−0.0281 (16)	−0.0042 (17)	−0.0098 (17)
C76B	0.0553 (17)	0.071 (2)	0.085 (3)	−0.0293 (16)	−0.0044 (18)	−0.0177 (19)
C77B	0.0481 (15)	0.0488 (16)	0.085 (2)	−0.0231 (13)	0.0071 (15)	−0.0217 (16)
C78B	0.0390 (13)	0.0400 (14)	0.077 (2)	−0.0169 (11)	0.0201 (13)	−0.0193 (13)
C79B	0.0581 (19)	0.081 (3)	0.076 (2)	−0.0287 (18)	0.0148 (18)	0.002 (2)
C80B	0.069 (2)	0.061 (2)	0.083 (3)	−0.0345 (17)	0.0098 (19)	−0.0035 (18)
N1S	0.053 (2)	0.093 (3)	0.218 (6)	−0.014 (2)	0.029 (3)	0.039 (4)
C11S	0.0584 (19)	0.0479 (17)	0.092 (3)	−0.0198 (15)	0.0169 (19)	0.0044 (17)
C12S	0.0449 (13)	0.0344 (12)	0.0497 (15)	−0.0187 (10)	0.0026 (11)	−0.0105 (11)
N2S	0.0559 (16)	0.069 (2)	0.100 (3)	−0.0175 (15)	−0.0169 (17)	−0.0164 (18)
C21S	0.0477 (14)	0.0487 (15)	0.0674 (19)	−0.0246 (12)	−0.0011 (14)	−0.0170 (14)
C22S	0.0470 (13)	0.0414 (13)	0.0541 (16)	−0.0191 (11)	−0.0015 (12)	−0.0121 (12)
N3S	0.099 (3)	0.228 (7)	0.094 (4)	−0.061 (4)	0.000 (3)	−0.002 (4)
C31S	0.0535 (19)	0.139 (4)	0.074 (3)	−0.042 (2)	0.0053 (19)	−0.018 (3)
C32S	0.0501 (18)	0.099 (3)	0.089 (3)	−0.024 (2)	−0.0007 (19)	−0.014 (2)
N4S	0.537 (8)	0.806 (13)	0.206 (11)	−0.596	0.109 (11)	−0.109 (12)
C41S	0.262 (8)	0.262 (9)	0.100 (5)	−0.203 (8)	0.018 (6)	−0.012 (6)
C42S	0.098 (5)	0.164 (8)	0.162 (8)	−0.003 (5)	−0.012 (5)	0.039 (6)

### Geometric parameters (Å, º)

**Table:**

S1A—O6A	1.439 (2)	Na1B—O5B	2.3686 (18)
S1A—O7A	1.449 (2)	Na1B—O2W	2.3854 (1)
S1A—N1A	1.604 (2)	Na1B—O10B	2.513 (3)
S1A—C55A	1.775 (3)	Na1B—Na2B	3.5967 (9)
S2A—O9A	1.442 (2)	Na2B—O8B	2.304 (2)
S2A—O10A	1.455 (2)	Na2B—O5B	2.3114 (19)
S2A—N3A	1.604 (2)	Na2B—O3B	2.3241 (17)
S2A—C69A	1.779 (2)	Na2B—O1B	2.3329 (18)
Na1A—O5A	2.2840 (18)	Na2B—O4B	2.352 (2)
Na1A—O8A	2.3344 (18)	Na2B—O2B	2.366 (2)
Na1A—O3A	2.3367 (17)	O1B—C1B	1.400 (3)
Na1A—O1A	2.3468 (17)	O1B—C53B	1.441 (2)
Na1A—O4A	2.357 (2)	O2B—C17B	1.391 (3)
Na1A—O2A	2.398 (2)	O2B—C45B	1.461 (4)
Na1A—Na2A	3.6001 (12)	O3B—C28B	1.395 (3)
Na2A—O1W	2.2692 (9)	O3B—C67B	1.434 (2)
Na2A—O8A	2.3481 (18)	O4B—C39B	1.393 (3)
Na2A—O7A	2.351 (2)	O4B—C49B	1.451 (3)
Na2A—O5A	2.3519 (19)	O5B—C54B	1.228 (3)
Na2A—O10A	2.399 (2)	O8B—C68B	1.234 (3)
O1A—C1A	1.395 (3)	O2W—H2W1	0.8306
O1A—C53A	1.435 (2)	O2W—H2W2	0.6805
O2A—C17A	1.397 (3)	N1B—C54B	1.342 (3)
O2A—C45A	1.437 (3)	N2B—C65B	1.405 (8)
O3A—C28A	1.397 (3)	N2B—C66B	1.436 (8)
O3A—C67A	1.443 (2)	N2B—C60B	1.469 (6)
O4A—C39A	1.398 (3)	N3B—C68B	1.335 (3)
O4A—C49A	1.470 (3)	N4B—C74B	1.440 (4)
O5A—C54A	1.241 (3)	N4B—C80B	1.454 (5)
O8A—C68A	1.245 (3)	N4B—C79B	1.464 (5)
O1W—H1W1	0.8943	C1B—C2B	1.391 (3)
O1W—H1W2	0.8636	C1B—C6B	1.396 (4)
N1A—C54A	1.325 (3)	C2B—C3B	1.406 (3)
N2A—C60A	1.410 (4)	C2B—C44B	1.517 (3)
N2A—C66A	1.440 (5)	C3B—C4B	1.391 (4)
N2A—C65A	1.449 (5)	C3B—H3BA	0.9500
N3A—C68A	1.336 (3)	C4B—C5B	1.390 (4)
N4A—C74A	1.426 (5)	C4B—C7B	1.542 (3)
N4A—C80A	1.453 (5)	C5B—C6B	1.403 (3)
N4A—C79A	1.501 (6)	C5B—H5BA	0.9500
C1A—C6A	1.384 (3)	C6B—C11B	1.520 (3)
C1A—C2A	1.401 (3)	C7B—C9B	1.521 (4)
C2A—C3A	1.395 (3)	C7B—C10B	1.527 (4)
C2A—C44A	1.518 (3)	C7B—C8B	1.528 (4)
C3A—C4A	1.399 (4)	C8B—H8BA	0.9800
C3A—H3AA	0.9500	C8B—H8BB	0.9800
C4A—C5A	1.392 (4)	C8B—H8BC	0.9800
C4A—C7A	1.535 (3)	C9B—H9BA	0.9800
C5A—C6A	1.401 (3)	C9B—H9BB	0.9800
C5A—H5AA	0.9500	C9B—H9BC	0.9800
C6A—C11A	1.520 (3)	C10B—H10D	0.9800
C7A—C9A	1.516 (5)	C10B—H10E	0.9800
C7A—C10A	1.520 (6)	C10B—H10F	0.9800
C7A—C8A	1.522 (4)	C11B—C12B	1.523 (3)
C8A—H8AA	0.9800	C11B—H11C	0.9900
C8A—H8AB	0.9800	C11B—H11D	0.9900
C8A—H8AC	0.9800	C12B—C17B	1.392 (4)
C9A—H9AA	0.9800	C12B—C13B	1.404 (4)
C9A—H9AB	0.9800	C13B—C14B	1.390 (4)
C9A—H9AC	0.9800	C13B—H13B	0.9500
C10A—H10A	0.9800	C14B—C15B	1.400 (4)
C10A—H10B	0.9800	C14B—C18B	1.529 (4)
C10A—H10C	0.9800	C15B—C16B	1.385 (4)
C11A—C12A	1.520 (3)	C15B—H15B	0.9500
C11A—H11A	0.9900	C16B—C17B	1.413 (3)
C11A—H11B	0.9900	C16B—C22B	1.524 (3)
C12A—C13A	1.391 (3)	C18B—C20B	1.520 (5)
C12A—C17A	1.400 (3)	C18B—C21B	1.531 (4)
C13A—C14A	1.394 (4)	C18B—C19B	1.542 (5)
C13A—H13A	0.9500	C19B—H19D	0.9800
C14A—C15A	1.387 (4)	C19B—H19E	0.9800
C14A—C18A	1.540 (3)	C19B—H19F	0.9800
C15A—C16A	1.398 (4)	C20B—H20D	0.9800
C15A—H15A	0.9500	C20B—H20E	0.9800
C16A—C17A	1.398 (3)	C20B—H20F	0.9800
C16A—C22A	1.534 (4)	C21B—H21D	0.9800
C18A—C19A	1.523 (5)	C21B—H21E	0.9800
C18A—C20A	1.530 (4)	C21B—H21F	0.9800
C18A—C21A	1.544 (5)	C22B—C23B	1.521 (3)
C19A—H19A	0.9800	C22B—H22C	0.9900
C19A—H19B	0.9800	C22B—H22D	0.9900
C19A—H19C	0.9800	C23B—C28B	1.390 (3)
C20A—H20A	0.9800	C23B—C24B	1.395 (3)
C20A—H20B	0.9800	C24B—C25B	1.391 (4)
C20A—H20C	0.9800	C24B—H24B	0.9500
C21A—H21A	0.9800	C25B—C26B	1.397 (4)
C21A—H21B	0.9800	C25B—C29B	1.536 (3)
C21A—H21C	0.9800	C26B—C27B	1.401 (3)
C22A—C23A	1.519 (4)	C26B—H26B	0.9500
C22A—H22A	0.9900	C27B—C28B	1.399 (3)
C22A—H22B	0.9900	C27B—C33B	1.520 (3)
C23A—C24A	1.397 (4)	C29B—C32B	1.516 (5)
C23A—C28A	1.398 (3)	C29B—C31B	1.535 (4)
C24A—C25A	1.384 (4)	C29B—C30B	1.543 (4)
C24A—H24A	0.9500	C30B—H30D	0.9800
C25A—C26A	1.393 (4)	C30B—H30E	0.9800
C25A—C29A	1.537 (4)	C30B—H30F	0.9800
C26A—C27A	1.399 (3)	C31B—H31D	0.9800
C26A—H26A	0.9500	C31B—H31E	0.9800
C27A—C28A	1.395 (4)	C31B—H31F	0.9800
C27A—C33A	1.518 (3)	C32B—H32D	0.9800
C29A—C31A	1.521 (5)	C32B—H32E	0.9800
C29A—C30A	1.538 (6)	C32B—H32F	0.9800
C29A—C32A	1.538 (6)	C33B—C34B	1.521 (3)
C30A—H30A	0.9800	C33B—H33C	0.9900
C30A—H30B	0.9800	C33B—H33D	0.9900
C30A—H30C	0.9800	C34B—C35B	1.388 (3)
C31A—H31A	0.9800	C34B—C39B	1.402 (3)
C31A—H31B	0.9800	C35B—C36B	1.404 (4)
C31A—H31C	0.9800	C35B—H35B	0.9500
C32A—H32A	0.9800	C36B—C37B	1.390 (4)
C32A—H32B	0.9800	C36B—C40B	1.535 (3)
C32A—H32C	0.9800	C37B—C38B	1.392 (3)
C33A—C34A	1.526 (3)	C37B—H37B	0.9500
C33A—H33A	0.9900	C38B—C39B	1.406 (3)
C33A—H33B	0.9900	C38B—C44B	1.522 (3)
C34A—C39A	1.389 (4)	C40B—C43B	1.515 (4)
C34A—C35A	1.400 (4)	C40B—C41B	1.530 (4)
C35A—C36A	1.398 (4)	C40B—C42B	1.533 (5)
C35A—H35A	0.9500	C41B—H41D	0.9800
C36A—C37A	1.386 (4)	C41B—H41E	0.9800
C36A—C40A	1.536 (4)	C41B—H41F	0.9800
C37A—C38A	1.388 (3)	C42B—H42D	0.9800
C37A—H37A	0.9500	C42B—H42E	0.9800
C38A—C39A	1.405 (3)	C42B—H42F	0.9800
C38A—C44A	1.534 (3)	C43B—H43D	0.9800
C40A—C41A	1.528 (5)	C43B—H43E	0.9800
C40A—C42A	1.529 (5)	C43B—H43F	0.9800
C40A—C43A	1.533 (5)	C44B—H44C	0.9900
C41A—H41A	0.9800	C44B—H44D	0.9900
C41A—H41B	0.9800	C45B—C46B	1.494 (4)
C41A—H41C	0.9800	C45B—H45C	0.9900
C42A—H42A	0.9800	C45B—H45D	0.9900
C42A—H42B	0.9800	C46B—C47B	1.513 (4)
C42A—H42C	0.9800	C46B—H46C	0.9900
C43A—H43A	0.9800	C46B—H46D	0.9900
C43A—H43B	0.9800	C47B—C48B	1.5100 (3)
C43A—H43C	0.9800	C47B—H47C	0.9900
C44A—H44A	0.9900	C47B—H47D	0.9900
C44A—H44B	0.9900	C48B—H48D	0.9800
C45A—C46A	1.494 (3)	C48B—H48E	0.9800
C45A—H45A	0.9900	C48B—H48F	0.9800
C45A—H45B	0.9900	C49B—C50B	1.493 (3)
C46A—C47A	1.509 (3)	C49B—H49C	0.9900
C46A—H46A	0.9900	C49B—H49D	0.9900
C46A—H46B	0.9900	C50B—C51B	1.480 (3)
C47A—C48A	1.479 (4)	C50B—H50C	0.9900
C47A—H47A	0.9900	C50B—H50D	0.9900
C47A—H47B	0.9900	C51B—C52B	1.527 (4)
C48A—H48A	0.9800	C51B—H51C	0.9900
C48A—H48B	0.9800	C51B—H51D	0.9900
C48A—H48C	0.9800	C52B—H52D	0.9800
C49A—C50A	1.489 (3)	C52B—H52E	0.9800
C49A—H49A	0.9900	C52B—H52F	0.9800
C49A—H49B	0.9900	C53B—C54B	1.525 (3)
C50A—C51A	1.511 (3)	C53B—H53C	0.9900
C50A—H50A	0.9900	C53B—H53D	0.9900
C50A—H50B	0.9900	C55B—C56B	1.369 (5)
C51A—C52A	1.519 (4)	C55B—C64B	1.426 (5)
C51A—H51A	0.9900	C56B—C57B	1.431 (7)
C51A—H51B	0.9900	C56B—H56B	0.9500
C52A—H52A	0.9800	C57B—C58B	1.339 (7)
C52A—H52B	0.9800	C57B—H57B	0.9500
C52A—H52C	0.9800	C58B—C59B	1.422 (6)
C53A—C54A	1.525 (3)	C58B—H58B	0.9500
C53A—H53A	0.9900	C59B—C60B	1.412 (6)
C53A—H53B	0.9900	C59B—C64B	1.434 (6)
C55A—C56A	1.359 (4)	C60B—C61B	1.376 (6)
C55A—C64A	1.430 (4)	C61B—C62B	1.423 (7)
C56A—C57A	1.417 (4)	C61B—H61B	0.9500
C56A—H56A	0.9500	C62B—C63B	1.357 (6)
C57A—C58A	1.358 (4)	C62B—H62B	0.9500
C57A—H57A	0.9500	C63B—C64B	1.418 (5)
C58A—C59A	1.410 (4)	C63B—H63B	0.9500
C58A—H58A	0.9500	C65B—H65D	0.9800
C59A—C64A	1.432 (4)	C65B—H65E	0.9800
C59A—C60A	1.445 (4)	C65B—H65F	0.9800
C60A—C61A	1.373 (4)	C66B—H66D	0.9800
C61A—C62A	1.394 (5)	C66B—H66E	0.9800
C61A—H61A	0.9500	C66B—H66F	0.9800
C62A—C63A	1.368 (5)	C67B—C68B	1.507 (3)
C62A—H62A	0.9500	C67B—H67C	0.9900
C63A—C64A	1.421 (4)	C67B—H67D	0.9900
C63A—H63A	0.9500	C69B—C70B	1.355 (5)
C65A—H65A	0.9800	C69B—C78B	1.434 (5)
C65A—H65B	0.9800	C70B—C71B	1.414 (5)
C65A—H65C	0.9800	C70B—H70B	0.9500
C66A—H66A	0.9800	C71B—C72B	1.366 (5)
C66A—H66B	0.9800	C71B—H71B	0.9500
C66A—H66C	0.9800	C72B—C73B	1.405 (5)
C67A—C68A	1.518 (3)	C72B—H72B	0.9500
C67A—H67A	0.9900	C73B—C74B	1.437 (5)
C67A—H67B	0.9900	C73B—C78B	1.438 (4)
C69A—C70A	1.367 (3)	C74B—C75B	1.357 (5)
C69A—C78A	1.425 (4)	C75B—C76B	1.416 (6)
C70A—C71A	1.415 (3)	C75B—H75B	0.9500
C70A—H70A	0.9500	C76B—C77B	1.360 (6)
C71A—C72A	1.369 (4)	C76B—H76B	0.9500
C71A—H71A	0.9500	C77B—C78B	1.422 (5)
C72A—C73A	1.413 (4)	C77B—H77B	0.9500
C72A—H72A	0.9500	C79B—H79D	0.9800
C73A—C78A	1.420 (4)	C79B—H79E	0.9800
C73A—C74A	1.457 (4)	C79B—H79F	0.9800
C74A—C75A	1.358 (6)	C80B—H80D	0.9800
C75A—C76A	1.389 (6)	C80B—H80E	0.9800
C75A—H75A	0.9500	C80B—H80F	0.9800
C76A—C77A	1.356 (5)	N1S—C11S	1.149 (5)
C76A—H76A	0.9500	C11S—C12S	1.431 (5)
C77A—C78A	1.426 (4)	C12S—H12A	0.9800
C77A—H77A	0.9500	C12S—H12B	0.9800
C79A—H79A	0.9800	C12S—H12C	0.9800
C79A—H79B	0.9800	N2S—C21S	1.136 (4)
C79A—H79C	0.9800	C21S—C22S	1.443 (4)
C80A—H80A	0.9800	C22S—H22E	0.9800
C80A—H80B	0.9800	C22S—H22F	0.9800
C80A—H80C	0.9800	C22S—H22G	0.9800
S1B—O7B	1.427 (2)	N3S—C31S	1.137 (7)
S1B—O6B	1.450 (3)	C31S—C32S	1.415 (7)
S1B—N1B	1.601 (2)	C32S—H32G	0.9800
S1B—C55B	1.794 (4)	C32S—H32H	0.9800
S2B—O10B	1.437 (3)	C32S—H32I	0.9800
S2B—O9B	1.443 (4)	N4S—C41S	1.097 (15)
S2B—N3B	1.598 (2)	C41S—C42S	1.248 (12)
S2B—C69B	1.783 (3)	C42S—H42G	0.9800
S2B—Na1B	3.1780 (10)	C42S—H42H	0.9800
Na1B—O6B	2.273 (2)	C42S—H42I	0.9800
Na1B—O8B	2.2978 (18)		
			
O6A—S1A—O7A	114.79 (14)	N4A—C80A—H80B	109.5
O6A—S1A—N1A	111.52 (13)	H80A—C80A—H80B	109.5
O7A—S1A—N1A	113.59 (11)	N4A—C80A—H80C	109.5
O6A—S1A—C55A	107.52 (12)	H80A—C80A—H80C	109.5
O7A—S1A—C55A	106.72 (12)	H80B—C80A—H80C	109.5
N1A—S1A—C55A	101.49 (11)	O7B—S1B—O6B	115.24 (15)
O6A—S1A—Na2A	90.94 (9)	O7B—S1B—N1B	106.57 (14)
O7A—S1A—Na2A	40.52 (9)	O6B—S1B—N1B	114.62 (14)
N1A—S1A—Na2A	96.23 (8)	O7B—S1B—C55B	109.66 (17)
C55A—S1A—Na2A	147.21 (9)	O6B—S1B—C55B	106.87 (17)
O9A—S2A—O10A	114.90 (13)	N1B—S1B—C55B	103.17 (15)
O9A—S2A—N3A	110.93 (12)	O10B—S2B—O9B	113.79 (19)
O10A—S2A—N3A	114.01 (11)	O10B—S2B—N3B	113.29 (16)
O9A—S2A—C69A	107.05 (12)	O9B—S2B—N3B	112.71 (18)
O10A—S2A—C69A	108.12 (12)	O10B—S2B—C69B	108.21 (18)
N3A—S2A—C69A	100.55 (11)	O9B—S2B—C69B	107.46 (18)
O9A—S2A—Na2A	93.39 (8)	N3B—S2B—C69B	100.21 (13)
O10A—S2A—Na2A	39.96 (8)	O10B—S2B—Na1B	50.18 (12)
N3A—S2A—Na2A	94.49 (8)	O9B—S2B—Na1B	79.54 (13)
C69A—S2A—Na2A	148.01 (9)	N3B—S2B—Na1B	96.97 (9)
O5A—Na1A—O8A	79.29 (6)	C69B—S2B—Na1B	156.92 (12)
O5A—Na1A—O3A	148.95 (7)	O6B—Na1B—O8B	153.12 (8)
O8A—Na1A—O3A	70.07 (6)	O6B—Na1B—O5B	75.86 (7)
O5A—Na1A—O1A	69.01 (6)	O8B—Na1B—O5B	77.40 (6)
O8A—Na1A—O1A	148.24 (7)	O6B—Na1B—O2W	92.22 (7)
O3A—Na1A—O1A	141.18 (6)	O8B—Na1B—O2W	104.06 (7)
O5A—Na1A—O4A	105.22 (7)	O5B—Na1B—O2W	121.68 (7)
O8A—Na1A—O4A	107.72 (7)	O6B—Na1B—O10B	123.18 (9)
O3A—Na1A—O4A	89.12 (7)	O8B—Na1B—O10B	74.18 (7)
O1A—Na1A—O4A	83.18 (7)	O5B—Na1B—O10B	131.35 (9)
O5A—Na1A—O2A	103.29 (7)	O2W—Na1B—O10B	103.20 (9)
O8A—Na1A—O2A	99.18 (7)	O6B—Na1B—S2B	142.94 (6)
O3A—Na1A—O2A	77.49 (6)	O8B—Na1B—S2B	61.34 (5)
O1A—Na1A—O2A	86.58 (6)	O5B—Na1B—S2B	136.16 (5)
O4A—Na1A—O2A	143.74 (7)	O2W—Na1B—S2B	84.39 (2)
O5A—Na1A—C68A	98.69 (6)	O10B—Na1B—S2B	26.05 (8)
O8A—Na1A—C68A	20.58 (6)	O6B—Na1B—Na2B	114.93 (6)
O3A—Na1A—C68A	50.30 (6)	O8B—Na1B—Na2B	38.65 (5)
O1A—Na1A—C68A	165.01 (7)	O5B—Na1B—Na2B	39.20 (5)
O4A—Na1A—C68A	109.04 (7)	O2W—Na1B—Na2B	115.00 (4)
O2A—Na1A—C68A	88.11 (6)	O10B—Na1B—Na2B	107.06 (6)
O5A—Na1A—Na2A	39.74 (5)	S2B—Na1B—Na2B	99.68 (2)
O8A—Na1A—Na2A	39.89 (4)	O8B—Na2B—O5B	78.44 (7)
O3A—Na1A—Na2A	109.24 (5)	O8B—Na2B—O3B	68.83 (6)
O1A—Na1A—Na2A	108.37 (5)	O5B—Na2B—O3B	145.74 (7)
O4A—Na1A—Na2A	115.62 (5)	O8B—Na2B—O1B	146.22 (7)
O2A—Na1A—Na2A	100.62 (5)	O5B—Na2B—O1B	69.83 (6)
C68A—Na1A—Na2A	58.95 (4)	O3B—Na2B—O1B	144.19 (7)
O1W—Na2A—O8A	100.32 (10)	O8B—Na2B—O4B	110.89 (9)
O1W—Na2A—O7A	113.50 (11)	O5B—Na2B—O4B	96.09 (8)
O8A—Na2A—O7A	144.59 (7)	O3B—Na2B—O4B	86.63 (7)
O1W—Na2A—O5A	126.12 (6)	O1B—Na2B—O4B	84.30 (7)
O8A—Na2A—O5A	77.65 (6)	O8B—Na2B—O2B	94.84 (8)
O7A—Na2A—O5A	74.19 (7)	O5B—Na2B—O2B	108.30 (8)
O1W—Na2A—O10A	86.34 (7)	O3B—Na2B—O2B	84.83 (7)
O8A—Na2A—O10A	76.91 (6)	O1B—Na2B—O2B	84.63 (7)
O7A—Na2A—O10A	114.11 (8)	O4B—Na2B—O2B	147.70 (7)
O5A—Na2A—O10A	141.80 (7)	O8B—Na2B—Na1B	38.53 (5)
O1W—Na2A—S1A	104.55 (8)	O5B—Na2B—Na1B	40.37 (5)
O8A—Na2A—S1A	138.21 (5)	O3B—Na2B—Na1B	107.32 (5)
O7A—Na2A—S1A	23.60 (5)	O1B—Na2B—Na1B	108.20 (5)
O5A—Na2A—S1A	60.56 (5)	O4B—Na2B—Na1B	112.11 (5)
O10A—Na2A—S1A	137.12 (6)	O2B—Na2B—Na1B	100.17 (5)
O1W—Na2A—S2A	76.12 (6)	C1B—O1B—C53B	111.74 (17)
O8A—Na2A—S2A	59.85 (4)	C1B—O1B—Na2B	130.00 (12)
O7A—Na2A—S2A	136.97 (6)	C53B—O1B—Na2B	118.21 (14)
O5A—Na2A—S2A	135.92 (5)	C17B—O2B—C45B	109.8 (2)
O10A—Na2A—S2A	22.92 (5)	C17B—O2B—Na2B	126.71 (15)
S1A—Na2A—S2A	159.34 (4)	C45B—O2B—Na2B	123.5 (2)
O1W—Na2A—Na1A	123.42 (8)	C28B—O3B—C67B	111.77 (16)
O8A—Na2A—Na1A	39.61 (4)	C28B—O3B—Na2B	128.60 (13)
O7A—Na2A—Na1A	108.28 (6)	C67B—O3B—Na2B	119.62 (13)
O5A—Na2A—Na1A	38.38 (4)	C39B—O4B—C49B	113.46 (19)
O10A—Na2A—Na1A	109.57 (5)	C39B—O4B—Na2B	127.68 (14)
S1A—Na2A—Na1A	98.70 (3)	C49B—O4B—Na2B	117.24 (14)
S2A—Na2A—Na1A	97.84 (3)	C54B—O5B—Na2B	121.32 (16)
O1W—Na2A—H1W1	18.8	C54B—O5B—Na1B	133.30 (16)
O8A—Na2A—H1W1	110.5	Na2B—O5B—Na1B	100.44 (7)
O7A—Na2A—H1W1	99.6	S1B—O6B—Na1B	131.76 (13)
O5A—Na2A—H1W1	111.4	C68B—O8B—Na1B	134.79 (17)
O10A—Na2A—H1W1	104.0	C68B—O8B—Na2B	121.47 (16)
S1A—Na2A—H1W1	87.0	Na1B—O8B—Na2B	102.82 (8)
S2A—Na2A—H1W1	95.0	S2B—O10B—Na1B	103.78 (17)
Na1A—Na2A—H1W1	121.3	Na1B—O2W—H2W1	108.2
O1W—Na2A—H1W2	18.0	Na1B—O2W—H2W2	116.8
O8A—Na2A—H1W2	113.1	H2W1—O2W—H2W2	133.5
O7A—Na2A—H1W2	102.3	C54B—N1B—S1B	120.2 (2)
O5A—Na2A—H1W2	139.0	C65B—N2B—C66B	109.5 (5)
O10A—Na2A—H1W2	77.8	C65B—N2B—C60B	113.6 (5)
S1A—Na2A—H1W2	99.9	C66B—N2B—C60B	117.6 (5)
S2A—Na2A—H1W2	74.2	C68B—N3B—S2B	118.4 (2)
Na1A—Na2A—H1W2	141.2	C74B—N4B—C80B	115.5 (3)
H1W1—Na2A—H1W2	27.8	C74B—N4B—C79B	111.5 (3)
C1A—O1A—C53A	111.71 (16)	C80B—N4B—C79B	112.1 (3)
C1A—O1A—Na1A	128.69 (12)	C2B—C1B—C6B	121.9 (2)
C53A—O1A—Na1A	119.24 (12)	C2B—C1B—O1B	119.0 (2)
C17A—O2A—C45A	114.1 (2)	C6B—C1B—O1B	119.1 (2)
C17A—O2A—Na1A	132.34 (15)	C1B—C2B—C3B	118.1 (2)
C45A—O2A—Na1A	113.04 (16)	C1B—C2B—C44B	122.3 (2)
C28A—O3A—C67A	109.64 (16)	C3B—C2B—C44B	119.6 (2)
C28A—O3A—Na1A	132.67 (13)	C4B—C3B—C2B	121.9 (2)
C67A—O3A—Na1A	117.62 (12)	C4B—C3B—H3BA	119.0
C39A—O4A—C49A	107.9 (2)	C2B—C3B—H3BA	119.0
C39A—O4A—Na1A	125.11 (14)	C5B—C4B—C3B	117.9 (2)
C49A—O4A—Na1A	126.96 (16)	C5B—C4B—C7B	120.3 (2)
C54A—O5A—Na1A	122.86 (14)	C3B—C4B—C7B	121.8 (2)
C54A—O5A—Na2A	134.09 (15)	C4B—C5B—C6B	122.3 (2)
Na1A—O5A—Na2A	101.89 (7)	C4B—C5B—H5BA	118.8
S1A—O7A—Na2A	115.87 (12)	C6B—C5B—H5BA	118.8
C68A—O8A—Na1A	118.18 (14)	C1B—C6B—C5B	117.7 (2)
C68A—O8A—Na2A	133.82 (15)	C1B—C6B—C11B	121.8 (2)
Na1A—O8A—Na2A	100.50 (7)	C5B—C6B—C11B	120.4 (2)
S2A—O10A—Na2A	117.12 (12)	C9B—C7B—C10B	107.4 (3)
Na2A—O1W—H1W1	106.1	C9B—C7B—C8B	109.2 (3)
Na2A—O1W—H1W2	107.5	C10B—C7B—C8B	109.1 (3)
H1W1—O1W—H1W2	93.1	C9B—C7B—C4B	112.0 (2)
C54A—N1A—S1A	119.33 (17)	C10B—C7B—C4B	108.5 (2)
C60A—N2A—C66A	116.4 (3)	C8B—C7B—C4B	110.5 (2)
C60A—N2A—C65A	117.5 (3)	C7B—C8B—H8BA	109.5
C66A—N2A—C65A	111.4 (3)	C7B—C8B—H8BB	109.5
C68A—N3A—S2A	119.03 (17)	H8BA—C8B—H8BB	109.5
C74A—N4A—C80A	115.4 (4)	C7B—C8B—H8BC	109.5
C74A—N4A—C79A	109.9 (4)	H8BA—C8B—H8BC	109.5
C80A—N4A—C79A	107.6 (4)	H8BB—C8B—H8BC	109.5
C6A—C1A—O1A	119.7 (2)	C7B—C9B—H9BA	109.5
C6A—C1A—C2A	121.7 (2)	C7B—C9B—H9BB	109.5
O1A—C1A—C2A	118.5 (2)	H9BA—C9B—H9BB	109.5
C3A—C2A—C1A	118.0 (2)	C7B—C9B—H9BC	109.5
C3A—C2A—C44A	120.0 (2)	H9BA—C9B—H9BC	109.5
C1A—C2A—C44A	121.8 (2)	H9BB—C9B—H9BC	109.5
C2A—C3A—C4A	122.0 (2)	C7B—C10B—H10D	109.5
C2A—C3A—H3AA	119.0	C7B—C10B—H10E	109.5
C4A—C3A—H3AA	119.0	H10D—C10B—H10E	109.5
C5A—C4A—C3A	117.8 (2)	C7B—C10B—H10F	109.5
C5A—C4A—C7A	122.1 (2)	H10D—C10B—H10F	109.5
C3A—C4A—C7A	120.1 (2)	H10E—C10B—H10F	109.5
C4A—C5A—C6A	121.9 (2)	C6B—C11B—C12B	110.11 (18)
C4A—C5A—H5AA	119.0	C6B—C11B—H11C	109.6
C6A—C5A—H5AA	119.0	C12B—C11B—H11C	109.6
C1A—C6A—C5A	118.4 (2)	C6B—C11B—H11D	109.6
C1A—C6A—C11A	122.1 (2)	C12B—C11B—H11D	109.6
C5A—C6A—C11A	119.5 (2)	H11C—C11B—H11D	108.2
C9A—C7A—C10A	108.7 (3)	C17B—C12B—C13B	118.2 (2)
C9A—C7A—C8A	108.4 (3)	C17B—C12B—C11B	122.4 (2)
C10A—C7A—C8A	109.2 (3)	C13B—C12B—C11B	119.3 (2)
C9A—C7A—C4A	108.4 (3)	C14B—C13B—C12B	122.3 (2)
C10A—C7A—C4A	112.4 (3)	C14B—C13B—H13B	118.9
C8A—C7A—C4A	109.7 (2)	C12B—C13B—H13B	118.9
C7A—C8A—H8AA	109.5	C13B—C14B—C15B	117.5 (2)
C7A—C8A—H8AB	109.5	C13B—C14B—C18B	123.2 (2)
H8AA—C8A—H8AB	109.5	C15B—C14B—C18B	119.4 (2)
C7A—C8A—H8AC	109.5	C16B—C15B—C14B	122.6 (2)
H8AA—C8A—H8AC	109.5	C16B—C15B—H15B	118.7
H8AB—C8A—H8AC	109.5	C14B—C15B—H15B	118.7
C7A—C9A—H9AA	109.5	C15B—C16B—C17B	118.0 (2)
C7A—C9A—H9AB	109.5	C15B—C16B—C22B	119.4 (2)
H9AA—C9A—H9AB	109.5	C17B—C16B—C22B	122.5 (2)
C7A—C9A—H9AC	109.5	O2B—C17B—C12B	119.8 (2)
H9AA—C9A—H9AC	109.5	O2B—C17B—C16B	119.1 (2)
H9AB—C9A—H9AC	109.5	C12B—C17B—C16B	121.1 (2)
C7A—C10A—H10A	109.5	C20B—C18B—C14B	108.9 (2)
C7A—C10A—H10B	109.5	C20B—C18B—C21B	109.6 (3)
H10A—C10A—H10B	109.5	C14B—C18B—C21B	112.4 (2)
C7A—C10A—H10C	109.5	C20B—C18B—C19B	109.6 (3)
H10A—C10A—H10C	109.5	C14B—C18B—C19B	109.0 (3)
H10B—C10A—H10C	109.5	C21B—C18B—C19B	107.3 (3)
C12A—C11A—C6A	108.44 (19)	C18B—C19B—H19D	109.5
C12A—C11A—H11A	110.0	C18B—C19B—H19E	109.5
C6A—C11A—H11A	110.0	H19D—C19B—H19E	109.5
C12A—C11A—H11B	110.0	C18B—C19B—H19F	109.5
C6A—C11A—H11B	110.0	H19D—C19B—H19F	109.5
H11A—C11A—H11B	108.4	H19E—C19B—H19F	109.5
C13A—C12A—C17A	118.1 (2)	C18B—C20B—H20D	109.5
C13A—C12A—C11A	119.6 (2)	C18B—C20B—H20E	109.5
C17A—C12A—C11A	122.0 (2)	H20D—C20B—H20E	109.5
C12A—C13A—C14A	122.5 (2)	C18B—C20B—H20F	109.5
C12A—C13A—H13A	118.7	H20D—C20B—H20F	109.5
C14A—C13A—H13A	118.7	H20E—C20B—H20F	109.5
C15A—C14A—C13A	117.5 (2)	C18B—C21B—H21D	109.5
C15A—C14A—C18A	123.5 (2)	C18B—C21B—H21E	109.5
C13A—C14A—C18A	119.0 (2)	H21D—C21B—H21E	109.5
C14A—C15A—C16A	122.3 (2)	C18B—C21B—H21F	109.5
C14A—C15A—H15A	118.8	H21D—C21B—H21F	109.5
C16A—C15A—H15A	118.8	H21E—C21B—H21F	109.5
C15A—C16A—C17A	118.2 (2)	C23B—C22B—C16B	110.41 (19)
C15A—C16A—C22A	120.6 (2)	C23B—C22B—H22C	109.6
C17A—C16A—C22A	121.2 (2)	C16B—C22B—H22C	109.6
O2A—C17A—C16A	119.8 (2)	C23B—C22B—H22D	109.6
O2A—C17A—C12A	119.1 (2)	C16B—C22B—H22D	109.6
C16A—C17A—C12A	121.1 (2)	H22C—C22B—H22D	108.1
C19A—C18A—C20A	110.0 (3)	C28B—C23B—C24B	118.2 (2)
C19A—C18A—C14A	108.5 (3)	C28B—C23B—C22B	121.3 (2)
C20A—C18A—C14A	112.2 (3)	C24B—C23B—C22B	120.4 (2)
C19A—C18A—C21A	108.5 (3)	C25B—C24B—C23B	122.0 (2)
C20A—C18A—C21A	107.6 (3)	C25B—C24B—H24B	119.0
C14A—C18A—C21A	109.9 (2)	C23B—C24B—H24B	119.0
C18A—C19A—H19A	109.5	C24B—C25B—C26B	118.0 (2)
C18A—C19A—H19B	109.5	C24B—C25B—C29B	122.9 (2)
H19A—C19A—H19B	109.5	C26B—C25B—C29B	119.2 (2)
C18A—C19A—H19C	109.5	C25B—C26B—C27B	122.1 (2)
H19A—C19A—H19C	109.5	C25B—C26B—H26B	118.9
H19B—C19A—H19C	109.5	C27B—C26B—H26B	118.9
C18A—C20A—H20A	109.5	C28B—C27B—C26B	117.4 (2)
C18A—C20A—H20B	109.5	C28B—C27B—C33B	122.08 (19)
H20A—C20A—H20B	109.5	C26B—C27B—C33B	120.4 (2)
C18A—C20A—H20C	109.5	C23B—C28B—O3B	118.9 (2)
H20A—C20A—H20C	109.5	C23B—C28B—C27B	122.1 (2)
H20B—C20A—H20C	109.5	O3B—C28B—C27B	119.0 (2)
C18A—C21A—H21A	109.5	C32B—C29B—C31B	108.1 (3)
C18A—C21A—H21B	109.5	C32B—C29B—C25B	109.7 (2)
H21A—C21A—H21B	109.5	C31B—C29B—C25B	111.7 (2)
C18A—C21A—H21C	109.5	C32B—C29B—C30B	112.1 (3)
H21A—C21A—H21C	109.5	C31B—C29B—C30B	106.7 (2)
H21B—C21A—H21C	109.5	C25B—C29B—C30B	108.6 (2)
C23A—C22A—C16A	112.5 (2)	C29B—C30B—H30D	109.5
C23A—C22A—H22A	109.1	C29B—C30B—H30E	109.5
C16A—C22A—H22A	109.1	H30D—C30B—H30E	109.5
C23A—C22A—H22B	109.1	C29B—C30B—H30F	109.5
C16A—C22A—H22B	109.1	H30D—C30B—H30F	109.5
H22A—C22A—H22B	107.8	H30E—C30B—H30F	109.5
C24A—C23A—C28A	117.7 (2)	C29B—C31B—H31D	109.5
C24A—C23A—C22A	120.6 (2)	C29B—C31B—H31E	109.5
C28A—C23A—C22A	121.7 (2)	H31D—C31B—H31E	109.5
C25A—C24A—C23A	122.6 (2)	C29B—C31B—H31F	109.5
C25A—C24A—H24A	118.7	H31D—C31B—H31F	109.5
C23A—C24A—H24A	118.7	H31E—C31B—H31F	109.5
C24A—C25A—C26A	117.7 (2)	C29B—C32B—H32D	109.5
C24A—C25A—C29A	122.9 (3)	C29B—C32B—H32E	109.5
C26A—C25A—C29A	119.4 (3)	H32D—C32B—H32E	109.5
C25A—C26A—C27A	122.3 (3)	C29B—C32B—H32F	109.5
C25A—C26A—H26A	118.9	H32D—C32B—H32F	109.5
C27A—C26A—H26A	118.9	H32E—C32B—H32F	109.5
C28A—C27A—C26A	117.8 (2)	C27B—C33B—C34B	109.63 (19)
C28A—C27A—C33A	121.9 (2)	C27B—C33B—H33C	109.7
C26A—C27A—C33A	120.1 (2)	C34B—C33B—H33C	109.7
C27A—C28A—O3A	119.0 (2)	C27B—C33B—H33D	109.7
C27A—C28A—C23A	121.7 (2)	C34B—C33B—H33D	109.7
O3A—C28A—C23A	119.3 (2)	H33C—C33B—H33D	108.2
C31A—C29A—C25A	108.4 (3)	C35B—C34B—C39B	118.1 (2)
C31A—C29A—C30A	108.9 (3)	C35B—C34B—C33B	119.8 (2)
C25A—C29A—C30A	111.0 (3)	C39B—C34B—C33B	121.9 (2)
C31A—C29A—C32A	109.7 (4)	C34B—C35B—C36B	122.9 (2)
C25A—C29A—C32A	109.6 (3)	C34B—C35B—H35B	118.6
C30A—C29A—C32A	109.3 (4)	C36B—C35B—H35B	118.6
C29A—C30A—H30A	109.5	C37B—C36B—C35B	116.8 (2)
C29A—C30A—H30B	109.5	C37B—C36B—C40B	123.3 (2)
H30A—C30A—H30B	109.5	C35B—C36B—C40B	119.9 (2)
C29A—C30A—H30C	109.5	C36B—C37B—C38B	122.9 (2)
H30A—C30A—H30C	109.5	C36B—C37B—H37B	118.6
H30B—C30A—H30C	109.5	C38B—C37B—H37B	118.6
C29A—C31A—H31A	109.5	C37B—C38B—C39B	118.1 (2)
C29A—C31A—H31B	109.5	C37B—C38B—C44B	120.0 (2)
H31A—C31A—H31B	109.5	C39B—C38B—C44B	121.7 (2)
C29A—C31A—H31C	109.5	O4B—C39B—C34B	119.4 (2)
H31A—C31A—H31C	109.5	O4B—C39B—C38B	119.79 (19)
H31B—C31A—H31C	109.5	C34B—C39B—C38B	120.8 (2)
C29A—C32A—H32A	109.5	C43B—C40B—C41B	108.8 (3)
C29A—C32A—H32B	109.5	C43B—C40B—C42B	109.7 (3)
H32A—C32A—H32B	109.5	C41B—C40B—C42B	108.0 (3)
C29A—C32A—H32C	109.5	C43B—C40B—C36B	110.6 (2)
H32A—C32A—H32C	109.5	C41B—C40B—C36B	111.2 (2)
H32B—C32A—H32C	109.5	C42B—C40B—C36B	108.5 (2)
C27A—C33A—C34A	109.19 (19)	C40B—C41B—H41D	109.5
C27A—C33A—H33A	109.8	C40B—C41B—H41E	109.5
C34A—C33A—H33A	109.8	H41D—C41B—H41E	109.5
C27A—C33A—H33B	109.8	C40B—C41B—H41F	109.5
C34A—C33A—H33B	109.8	H41D—C41B—H41F	109.5
H33A—C33A—H33B	108.3	H41E—C41B—H41F	109.5
C39A—C34A—C35A	117.8 (2)	C40B—C42B—H42D	109.5
C39A—C34A—C33A	122.7 (2)	C40B—C42B—H42E	109.5
C35A—C34A—C33A	119.3 (2)	H42D—C42B—H42E	109.5
C36A—C35A—C34A	122.3 (2)	C40B—C42B—H42F	109.5
C36A—C35A—H35A	118.9	H42D—C42B—H42F	109.5
C34A—C35A—H35A	118.9	H42E—C42B—H42F	109.5
C37A—C36A—C35A	117.5 (2)	C40B—C43B—H43D	109.5
C37A—C36A—C40A	120.2 (2)	C40B—C43B—H43E	109.5
C35A—C36A—C40A	122.3 (3)	H43D—C43B—H43E	109.5
C36A—C37A—C38A	122.6 (2)	C40B—C43B—H43F	109.5
C36A—C37A—H37A	118.7	H43D—C43B—H43F	109.5
C38A—C37A—H37A	118.7	H43E—C43B—H43F	109.5
C37A—C38A—C39A	118.0 (2)	C2B—C44B—C38B	110.85 (18)
C37A—C38A—C44A	119.8 (2)	C2B—C44B—H44C	109.5
C39A—C38A—C44A	122.1 (2)	C38B—C44B—H44C	109.5
C34A—C39A—O4A	119.6 (2)	C2B—C44B—H44D	109.5
C34A—C39A—C38A	121.6 (2)	C38B—C44B—H44D	109.5
O4A—C39A—C38A	118.8 (2)	H44C—C44B—H44D	108.1
C41A—C40A—C42A	108.1 (3)	O2B—C45B—C46B	116.0 (4)
C41A—C40A—C43A	108.5 (3)	O2B—C45B—H45C	108.3
C42A—C40A—C43A	108.7 (3)	C46B—C45B—H45C	108.3
C41A—C40A—C36A	108.8 (3)	O2B—C45B—H45D	108.3
C42A—C40A—C36A	113.0 (3)	C46B—C45B—H45D	108.3
C43A—C40A—C36A	109.6 (2)	H45C—C45B—H45D	107.4
C40A—C41A—H41A	109.5	C45B—C46B—C47B	110.9 (3)
C40A—C41A—H41B	109.5	C45B—C46B—H46C	109.5
H41A—C41A—H41B	109.5	C47B—C46B—H46C	109.5
C40A—C41A—H41C	109.5	C45B—C46B—H46D	109.5
H41A—C41A—H41C	109.5	C47B—C46B—H46D	109.5
H41B—C41A—H41C	109.5	H46C—C46B—H46D	108.1
C40A—C42A—H42A	109.5	C48B—C47B—C46B	109.4 (3)
C40A—C42A—H42B	109.5	C48B—C47B—H47C	109.8
H42A—C42A—H42B	109.5	C46B—C47B—H47C	109.8
C40A—C42A—H42C	109.5	C48B—C47B—H47D	109.8
H42A—C42A—H42C	109.5	C46B—C47B—H47D	109.8
H42B—C42A—H42C	109.5	H47C—C47B—H47D	108.3
C40A—C43A—H43A	109.5	C47B—C48B—H48D	109.5
C40A—C43A—H43B	109.5	C47B—C48B—H48E	109.5
H43A—C43A—H43B	109.5	H48D—C48B—H48E	109.5
C40A—C43A—H43C	109.5	C47B—C48B—H48F	109.5
H43A—C43A—H43C	109.5	H48D—C48B—H48F	109.5
H43B—C43A—H43C	109.5	H48E—C48B—H48F	109.5
C2A—C44A—C38A	109.74 (18)	O4B—C49B—C50B	114.4 (2)
C2A—C44A—H44A	109.7	O4B—C49B—H49C	108.7
C38A—C44A—H44A	109.7	C50B—C49B—H49C	108.7
C2A—C44A—H44B	109.7	O4B—C49B—H49D	108.7
C38A—C44A—H44B	109.7	C50B—C49B—H49D	108.7
H44A—C44A—H44B	108.2	H49C—C49B—H49D	107.6
O2A—C45A—C46A	112.9 (2)	C51B—C50B—C49B	115.0 (3)
O2A—C45A—H45A	109.0	C51B—C50B—H50C	108.5
C46A—C45A—H45A	109.0	C49B—C50B—H50C	108.5
O2A—C45A—H45B	109.0	C51B—C50B—H50D	108.5
C46A—C45A—H45B	109.0	C49B—C50B—H50D	108.5
H45A—C45A—H45B	107.8	H50C—C50B—H50D	107.5
C45A—C46A—C47A	109.6 (3)	C50B—C51B—C52B	109.3 (3)
C45A—C46A—H46A	109.7	C50B—C51B—H51C	109.8
C47A—C46A—H46A	109.7	C52B—C51B—H51C	109.8
C45A—C46A—H46B	109.7	C50B—C51B—H51D	109.8
C47A—C46A—H46B	109.7	C52B—C51B—H51D	109.8
H46A—C46A—H46B	108.2	H51C—C51B—H51D	108.3
C48A—C47A—C46A	113.2 (3)	C51B—C52B—H52D	109.5
C48A—C47A—H47A	108.9	C51B—C52B—H52E	109.5
C46A—C47A—H47A	108.9	H52D—C52B—H52E	109.5
C48A—C47A—H47B	108.9	C51B—C52B—H52F	109.5
C46A—C47A—H47B	108.9	H52D—C52B—H52F	109.5
H47A—C47A—H47B	107.8	H52E—C52B—H52F	109.5
C47A—C48A—H48A	109.5	O1B—C53B—C54B	110.07 (19)
C47A—C48A—H48B	109.5	O1B—C53B—H53C	109.6
H48A—C48A—H48B	109.5	C54B—C53B—H53C	109.6
C47A—C48A—H48C	109.5	O1B—C53B—H53D	109.6
H48A—C48A—H48C	109.5	C54B—C53B—H53D	109.6
H48B—C48A—H48C	109.5	H53C—C53B—H53D	108.2
O4A—C49A—C50A	110.8 (2)	O5B—C54B—N1B	128.6 (2)
O4A—C49A—H49A	109.5	O5B—C54B—C53B	120.4 (2)
C50A—C49A—H49A	109.5	N1B—C54B—C53B	110.9 (2)
O4A—C49A—H49B	109.5	C56B—C55B—C64B	122.1 (4)
C50A—C49A—H49B	109.5	C56B—C55B—S1B	117.4 (3)
H49A—C49A—H49B	108.1	C64B—C55B—S1B	120.4 (3)
C49A—C50A—C51A	111.0 (3)	C55B—C56B—C57B	120.3 (4)
C49A—C50A—H50A	109.4	C55B—C56B—H56B	119.9
C51A—C50A—H50A	109.4	C57B—C56B—H56B	119.9
C49A—C50A—H50B	109.4	C58B—C57B—C56B	119.5 (4)
C51A—C50A—H50B	109.4	C58B—C57B—H57B	120.2
H50A—C50A—H50B	108.0	C56B—C57B—H57B	120.2
C50A—C51A—C52A	109.2 (3)	C57B—C58B—C59B	121.3 (5)
C50A—C51A—H51A	109.8	C57B—C58B—H58B	119.4
C52A—C51A—H51A	109.8	C59B—C58B—H58B	119.4
C50A—C51A—H51B	109.8	C60B—C59B—C58B	120.1 (4)
C52A—C51A—H51B	109.8	C60B—C59B—C64B	119.4 (4)
H51A—C51A—H51B	108.3	C58B—C59B—C64B	120.5 (4)
C51A—C52A—H52A	109.5	C61B—C60B—C59B	120.7 (4)
C51A—C52A—H52B	109.5	C61B—C60B—N2B	120.3 (4)
H52A—C52A—H52B	109.5	C59B—C60B—N2B	118.9 (4)
C51A—C52A—H52C	109.5	C60B—C61B—C62B	119.1 (4)
H52A—C52A—H52C	109.5	C60B—C61B—H61B	120.4
H52B—C52A—H52C	109.5	C62B—C61B—H61B	120.4
O1A—C53A—C54A	108.92 (17)	C63B—C62B—C61B	122.0 (4)
O1A—C53A—H53A	109.9	C63B—C62B—H62B	119.0
C54A—C53A—H53A	109.9	C61B—C62B—H62B	119.0
O1A—C53A—H53B	109.9	C62B—C63B—C64B	119.9 (4)
C54A—C53A—H53B	109.9	C62B—C63B—H63B	120.0
H53A—C53A—H53B	108.3	C64B—C63B—H63B	120.0
O5A—C54A—N1A	129.3 (2)	C63B—C64B—C55B	125.2 (4)
O5A—C54A—C53A	119.86 (19)	C63B—C64B—C59B	118.9 (3)
N1A—C54A—C53A	110.82 (19)	C55B—C64B—C59B	115.9 (3)
C56A—C55A—C64A	121.9 (2)	N2B—C65B—H65D	109.5
C56A—C55A—S1A	116.4 (2)	N2B—C65B—H65E	109.5
C64A—C55A—S1A	121.60 (18)	H65D—C65B—H65E	109.5
C55A—C56A—C57A	119.7 (3)	N2B—C65B—H65F	109.5
C55A—C56A—H56A	120.2	H65D—C65B—H65F	109.5
C57A—C56A—H56A	120.2	H65E—C65B—H65F	109.5
C58A—C57A—C56A	120.4 (2)	N2B—C66B—H66D	109.5
C58A—C57A—H57A	119.8	N2B—C66B—H66E	109.5
C56A—C57A—H57A	119.8	H66D—C66B—H66E	109.5
C57A—C58A—C59A	121.4 (2)	N2B—C66B—H66F	109.5
C57A—C58A—H58A	119.3	H66D—C66B—H66F	109.5
C59A—C58A—H58A	119.3	H66E—C66B—H66F	109.5
C58A—C59A—C64A	119.0 (3)	O3B—C67B—C68B	108.80 (18)
C58A—C59A—C60A	121.5 (3)	O3B—C67B—H67C	109.9
C64A—C59A—C60A	119.4 (2)	C68B—C67B—H67C	109.9
C61A—C60A—N2A	122.4 (3)	O3B—C67B—H67D	109.9
C61A—C60A—C59A	119.3 (3)	C68B—C67B—H67D	109.9
N2A—C60A—C59A	118.2 (3)	H67C—C67B—H67D	108.3
C60A—C61A—C62A	120.7 (3)	O8B—C68B—N3B	128.1 (2)
C60A—C61A—H61A	119.6	O8B—C68B—C67B	120.7 (2)
C62A—C61A—H61A	119.6	N3B—C68B—C67B	111.2 (2)
C63A—C62A—C61A	121.8 (3)	C70B—C69B—C78B	122.1 (3)
C63A—C62A—H62A	119.1	C70B—C69B—S2B	115.8 (3)
C61A—C62A—H62A	119.1	C78B—C69B—S2B	122.1 (2)
C62A—C63A—C64A	120.3 (3)	C69B—C70B—C71B	120.0 (3)
C62A—C63A—H63A	119.8	C69B—C70B—H70B	120.0
C64A—C63A—H63A	119.8	C71B—C70B—H70B	120.0
C63A—C64A—C55A	124.2 (2)	C72B—C71B—C70B	120.0 (3)
C63A—C64A—C59A	118.4 (3)	C72B—C71B—H71B	120.0
C55A—C64A—C59A	117.5 (2)	C70B—C71B—H71B	120.0
N2A—C65A—H65A	109.5	C71B—C72B—C73B	121.7 (3)
N2A—C65A—H65B	109.5	C71B—C72B—H72B	119.1
H65A—C65A—H65B	109.5	C73B—C72B—H72B	119.1
N2A—C65A—H65C	109.5	C72B—C73B—C74B	121.6 (3)
H65A—C65A—H65C	109.5	C72B—C73B—C78B	119.1 (3)
H65B—C65A—H65C	109.5	C74B—C73B—C78B	119.2 (3)
N2A—C66A—H66A	109.5	C75B—C74B—C73B	120.1 (3)
N2A—C66A—H66B	109.5	C75B—C74B—N4B	122.7 (3)
H66A—C66A—H66B	109.5	C73B—C74B—N4B	117.2 (3)
N2A—C66A—H66C	109.5	C74B—C75B—C76B	120.7 (4)
H66A—C66A—H66C	109.5	C74B—C75B—H75B	119.7
H66B—C66A—H66C	109.5	C76B—C75B—H75B	119.7
O3A—C67A—C68A	110.02 (17)	C77B—C76B—C75B	120.8 (4)
O3A—C67A—H67A	109.7	C77B—C76B—H76B	119.6
C68A—C67A—H67A	109.7	C75B—C76B—H76B	119.6
O3A—C67A—H67B	109.7	C76B—C77B—C78B	121.1 (3)
C68A—C67A—H67B	109.7	C76B—C77B—H77B	119.5
H67A—C67A—H67B	108.2	C78B—C77B—H77B	119.5
O8A—C68A—N3A	130.1 (2)	C77B—C78B—C69B	125.0 (3)
O8A—C68A—C67A	119.7 (2)	C77B—C78B—C73B	118.0 (3)
N3A—C68A—C67A	110.23 (19)	C69B—C78B—C73B	117.1 (3)
O8A—C68A—Na1A	41.23 (11)	N4B—C79B—H79D	109.5
N3A—C68A—Na1A	160.77 (16)	N4B—C79B—H79E	109.5
C67A—C68A—Na1A	81.61 (12)	H79D—C79B—H79E	109.5
C70A—C69A—C78A	122.0 (2)	N4B—C79B—H79F	109.5
C70A—C69A—S2A	117.11 (19)	H79D—C79B—H79F	109.5
C78A—C69A—S2A	120.88 (19)	H79E—C79B—H79F	109.5
C69A—C70A—C71A	119.1 (2)	N4B—C80B—H80D	109.5
C69A—C70A—H70A	120.5	N4B—C80B—H80E	109.5
C71A—C70A—H70A	120.5	H80D—C80B—H80E	109.5
C72A—C71A—C70A	120.5 (2)	N4B—C80B—H80F	109.5
C72A—C71A—H71A	119.8	H80D—C80B—H80F	109.5
C70A—C71A—H71A	119.8	H80E—C80B—H80F	109.5
C71A—C72A—C73A	121.4 (3)	N1S—C11S—C12S	178.4 (6)
C71A—C72A—H72A	119.3	C11S—C12S—H12A	109.5
C73A—C72A—H72A	119.3	C11S—C12S—H12B	109.5
C72A—C73A—C78A	118.7 (3)	H12A—C12S—H12B	109.5
C72A—C73A—C74A	122.6 (3)	C11S—C12S—H12C	109.5
C78A—C73A—C74A	118.7 (3)	H12A—C12S—H12C	109.5
C75A—C74A—N4A	123.7 (3)	H12B—C12S—H12C	109.5
C75A—C74A—C73A	119.7 (3)	N2S—C21S—C22S	178.6 (4)
N4A—C74A—C73A	116.6 (3)	C21S—C22S—H22E	109.5
C74A—C75A—C76A	120.9 (3)	C21S—C22S—H22F	109.5
C74A—C75A—H75A	119.5	H22E—C22S—H22F	109.5
C76A—C75A—H75A	119.5	C21S—C22S—H22G	109.5
C77A—C76A—C75A	121.5 (3)	H22E—C22S—H22G	109.5
C77A—C76A—H76A	119.3	H22F—C22S—H22G	109.5
C75A—C76A—H76A	119.3	N3S—C31S—C32S	177.9 (8)
C76A—C77A—C78A	120.9 (3)	C31S—C32S—H32G	109.5
C76A—C77A—H77A	119.5	C31S—C32S—H32H	109.5
C78A—C77A—H77A	119.5	H32G—C32S—H32H	109.5
C73A—C78A—C69A	118.2 (2)	C31S—C32S—H32I	109.5
C73A—C78A—C77A	118.2 (3)	H32G—C32S—H32I	109.5
C69A—C78A—C77A	123.5 (3)	H32H—C32S—H32I	109.5
N4A—C79A—H79A	109.5	N4S—C41S—C42S	155.7 (13)
N4A—C79A—H79B	109.5	C41S—C42S—H42G	109.5
H79A—C79A—H79B	109.5	C41S—C42S—H42H	109.5
N4A—C79A—H79C	109.5	H42G—C42S—H42H	109.5
H79A—C79A—H79C	109.5	C41S—C42S—H42I	109.5
H79B—C79A—H79C	109.5	H42G—C42S—H42I	109.5
N4A—C80A—H80A	109.5	H42H—C42S—H42I	109.5
			
O6A—S1A—Na2A—O1W	−13.01 (11)	O5A—Na1A—C68A—C67A	177.67 (13)
O7A—S1A—Na2A—O1W	115.76 (14)	O8A—Na1A—C68A—C67A	158.0 (2)
N1A—S1A—Na2A—O1W	−124.78 (11)	O3A—Na1A—C68A—C67A	−3.91 (12)
C55A—S1A—Na2A—O1W	112.56 (17)	O1A—Na1A—C68A—C67A	−148.4 (2)
O6A—S1A—Na2A—O8A	111.62 (12)	O4A—Na1A—C68A—C67A	68.18 (14)
O7A—S1A—Na2A—O8A	−119.61 (15)	O2A—Na1A—C68A—C67A	−79.18 (13)
N1A—S1A—Na2A—O8A	−0.15 (12)	Na2A—Na1A—C68A—C67A	177.23 (14)
C55A—S1A—Na2A—O8A	−122.81 (17)	O9A—S2A—C69A—C70A	4.1 (2)
O6A—S1A—Na2A—O7A	−128.77 (16)	O10A—S2A—C69A—C70A	128.36 (19)
N1A—S1A—Na2A—O7A	119.46 (16)	N3A—S2A—C69A—C70A	−111.9 (2)
C55A—S1A—Na2A—O7A	−3.2 (2)	Na2A—S2A—C69A—C70A	131.48 (17)
O6A—S1A—Na2A—O5A	110.56 (11)	O9A—S2A—C69A—C78A	−175.67 (19)
O7A—S1A—Na2A—O5A	−120.67 (14)	O10A—S2A—C69A—C78A	−51.4 (2)
N1A—S1A—Na2A—O5A	−1.21 (10)	N3A—S2A—C69A—C78A	68.4 (2)
C55A—S1A—Na2A—O5A	−123.88 (17)	Na2A—S2A—C69A—C78A	−48.2 (3)
O6A—S1A—Na2A—O10A	−113.52 (12)	C78A—C69A—C70A—C71A	−0.1 (4)
O7A—S1A—Na2A—O10A	15.25 (15)	S2A—C69A—C70A—C71A	−179.85 (18)
N1A—S1A—Na2A—O10A	134.71 (12)	C69A—C70A—C71A—C72A	1.2 (4)
C55A—S1A—Na2A—O10A	12.04 (18)	C70A—C71A—C72A—C73A	−0.4 (4)
O6A—S1A—Na2A—S2A	−102.19 (12)	C71A—C72A—C73A—C78A	−1.5 (4)
O7A—S1A—Na2A—S2A	26.59 (15)	C71A—C72A—C73A—C74A	179.7 (3)
N1A—S1A—Na2A—S2A	146.05 (11)	C80A—N4A—C74A—C75A	−16.6 (6)
C55A—S1A—Na2A—S2A	23.38 (19)	C79A—N4A—C74A—C75A	105.3 (5)
O6A—S1A—Na2A—Na1A	114.97 (9)	C80A—N4A—C74A—C73A	160.7 (4)
O7A—S1A—Na2A—Na1A	−116.26 (13)	C79A—N4A—C74A—C73A	−77.4 (5)
N1A—S1A—Na2A—Na1A	3.20 (9)	C72A—C73A—C74A—C75A	174.1 (3)
C55A—S1A—Na2A—Na1A	−119.46 (16)	C78A—C73A—C74A—C75A	−4.6 (5)
O9A—S2A—Na2A—O1W	11.31 (11)	C72A—C73A—C74A—N4A	−3.2 (5)
O10A—S2A—Na2A—O1W	−114.56 (15)	C78A—C73A—C74A—N4A	178.0 (3)
N3A—S2A—Na2A—O1W	122.63 (11)	N4A—C74A—C75A—C76A	−179.4 (4)
C69A—S2A—Na2A—O1W	−119.18 (17)	C73A—C74A—C75A—C76A	3.4 (6)
O9A—S2A—Na2A—O8A	−99.61 (10)	C74A—C75A—C76A—C77A	−0.8 (6)
O10A—S2A—Na2A—O8A	134.52 (14)	C75A—C76A—C77A—C78A	−0.7 (5)
N3A—S2A—Na2A—O8A	11.71 (10)	C72A—C73A—C78A—C69A	2.5 (4)
C69A—S2A—Na2A—O8A	129.90 (16)	C74A—C73A—C78A—C69A	−178.7 (3)
O9A—S2A—Na2A—O7A	121.01 (12)	C72A—C73A—C78A—C77A	−175.6 (3)
O10A—S2A—Na2A—O7A	−4.87 (15)	C74A—C73A—C78A—C77A	3.2 (4)
N3A—S2A—Na2A—O7A	−127.67 (12)	C70A—C69A—C78A—C73A	−1.8 (4)
C69A—S2A—Na2A—O7A	−9.48 (18)	S2A—C69A—C78A—C73A	177.95 (19)
O9A—S2A—Na2A—O5A	−116.84 (11)	C70A—C69A—C78A—C77A	176.3 (3)
O10A—S2A—Na2A—O5A	117.29 (15)	S2A—C69A—C78A—C77A	−4.0 (4)
N3A—S2A—Na2A—O5A	−5.51 (12)	C76A—C77A—C78A—C73A	−0.7 (4)
C69A—S2A—Na2A—O5A	112.68 (17)	C76A—C77A—C78A—C69A	−178.7 (3)
O9A—S2A—Na2A—O10A	125.87 (16)	O10B—S2B—Na1B—O6B	−50.1 (2)
N3A—S2A—Na2A—O10A	−122.81 (16)	O9B—S2B—Na1B—O6B	83.36 (18)
C69A—S2A—Na2A—O10A	−4.6 (2)	N3B—S2B—Na1B—O6B	−164.75 (17)
O9A—S2A—Na2A—S1A	105.78 (11)	C69B—S2B—Na1B—O6B	−26.8 (3)
O10A—S2A—Na2A—S1A	−20.09 (15)	O10B—S2B—Na1B—O8B	114.26 (17)
N3A—S2A—Na2A—S1A	−142.90 (11)	O9B—S2B—Na1B—O8B	−112.26 (14)
C69A—S2A—Na2A—S1A	−24.71 (18)	N3B—S2B—Na1B—O8B	−0.37 (14)
O9A—S2A—Na2A—Na1A	−111.28 (8)	C69B—S2B—Na1B—O8B	137.6 (3)
O10A—S2A—Na2A—Na1A	122.84 (13)	O10B—S2B—Na1B—O5B	92.38 (17)
N3A—S2A—Na2A—Na1A	0.04 (9)	O9B—S2B—Na1B—O5B	−134.14 (15)
C69A—S2A—Na2A—Na1A	118.23 (15)	N3B—S2B—Na1B—O5B	−22.25 (16)
O5A—Na1A—Na2A—O1W	107.72 (11)	C69B—S2B—Na1B—O5B	115.7 (3)
O8A—Na1A—Na2A—O1W	−62.58 (10)	O10B—S2B—Na1B—O2W	−136.36 (15)
O3A—Na1A—Na2A—O1W	−73.89 (9)	O9B—S2B—Na1B—O2W	−2.88 (13)
O1A—Na1A—Na2A—O1W	115.88 (9)	N3B—S2B—Na1B—O2W	109.01 (13)
O4A—Na1A—Na2A—O1W	24.71 (10)	C69B—S2B—Na1B—O2W	−113.1 (3)
O2A—Na1A—Na2A—O1W	−154.22 (9)	O9B—S2B—Na1B—O10B	133.5 (2)
C68A—Na1A—Na2A—O1W	−72.96 (9)	N3B—S2B—Na1B—O10B	−114.6 (2)
O5A—Na1A—Na2A—O8A	170.30 (11)	C69B—S2B—Na1B—O10B	23.3 (3)
O3A—Na1A—Na2A—O8A	−11.31 (8)	O10B—S2B—Na1B—Na2B	109.18 (15)
O1A—Na1A—Na2A—O8A	178.46 (9)	O9B—S2B—Na1B—Na2B	−117.34 (12)
O4A—Na1A—Na2A—O8A	87.28 (9)	N3B—S2B—Na1B—Na2B	−5.44 (13)
O2A—Na1A—Na2A—O8A	−91.64 (9)	C69B—S2B—Na1B—Na2B	132.5 (3)
C68A—Na1A—Na2A—O8A	−10.39 (8)	O6B—Na1B—Na2B—O8B	173.55 (14)
O5A—Na1A—Na2A—O7A	−28.42 (10)	O5B—Na1B—Na2B—O8B	168.66 (14)
O8A—Na1A—Na2A—O7A	161.28 (10)	O2W—Na1B—Na2B—O8B	−81.15 (11)
O3A—Na1A—Na2A—O7A	149.97 (8)	O10B—Na1B—Na2B—O8B	32.84 (14)
O1A—Na1A—Na2A—O7A	−20.26 (9)	S2B—Na1B—Na2B—O8B	7.13 (11)
O4A—Na1A—Na2A—O7A	−111.44 (8)	O6B—Na1B—Na2B—O5B	4.89 (13)
O2A—Na1A—Na2A—O7A	69.64 (8)	O8B—Na1B—Na2B—O5B	−168.66 (14)
C68A—Na1A—Na2A—O7A	150.89 (8)	O2W—Na1B—Na2B—O5B	110.19 (10)
O8A—Na1A—Na2A—O5A	−170.30 (11)	O10B—Na1B—Na2B—O5B	−135.82 (13)
O3A—Na1A—Na2A—O5A	178.39 (10)	S2B—Na1B—Na2B—O5B	−161.53 (10)
O1A—Na1A—Na2A—O5A	8.16 (9)	O6B—Na1B—Na2B—O3B	170.82 (10)
O4A—Na1A—Na2A—O5A	−83.02 (10)	O8B—Na1B—Na2B—O3B	−2.73 (11)
O2A—Na1A—Na2A—O5A	98.06 (9)	O5B—Na1B—Na2B—O3B	165.93 (12)
C68A—Na1A—Na2A—O5A	179.31 (10)	O2W—Na1B—Na2B—O3B	−83.88 (6)
O5A—Na1A—Na2A—O10A	−153.46 (10)	O10B—Na1B—Na2B—O3B	30.12 (10)
O8A—Na1A—Na2A—O10A	36.25 (9)	S2B—Na1B—Na2B—O3B	4.41 (6)
O3A—Na1A—Na2A—O10A	24.93 (8)	O6B—Na1B—Na2B—O1B	−13.83 (11)
O1A—Na1A—Na2A—O10A	−145.30 (8)	O8B—Na1B—Na2B—O1B	172.62 (12)
O4A—Na1A—Na2A—O10A	123.53 (8)	O5B—Na1B—Na2B—O1B	−18.72 (11)
O2A—Na1A—Na2A—O10A	−55.39 (8)	O2W—Na1B—Na2B—O1B	91.47 (6)
C68A—Na1A—Na2A—O10A	25.86 (8)	O10B—Na1B—Na2B—O1B	−154.53 (10)
O5A—Na1A—Na2A—S1A	−6.20 (8)	S2B—Na1B—Na2B—O1B	179.76 (6)
O8A—Na1A—Na2A—S1A	−176.50 (8)	O6B—Na1B—Na2B—O4B	77.37 (10)
O3A—Na1A—Na2A—S1A	172.19 (5)	O8B—Na1B—Na2B—O4B	−96.18 (12)
O1A—Na1A—Na2A—S1A	1.96 (6)	O5B—Na1B—Na2B—O4B	72.48 (11)
O4A—Na1A—Na2A—S1A	−89.22 (6)	O2W—Na1B—Na2B—O4B	−177.34 (5)
O2A—Na1A—Na2A—S1A	91.86 (5)	O10B—Na1B—Na2B—O4B	−63.34 (10)
C68A—Na1A—Na2A—S1A	173.11 (6)	S2B—Na1B—Na2B—O4B	−89.05 (6)
O5A—Na1A—Na2A—S2A	−173.78 (8)	O6B—Na1B—Na2B—O2B	−101.47 (10)
O8A—Na1A—Na2A—S2A	15.93 (7)	O8B—Na1B—Na2B—O2B	84.98 (12)
O3A—Na1A—Na2A—S2A	4.61 (6)	O5B—Na1B—Na2B—O2B	−106.36 (11)
O1A—Na1A—Na2A—S2A	−165.62 (5)	O2W—Na1B—Na2B—O2B	3.83 (6)
O4A—Na1A—Na2A—S2A	103.21 (6)	O10B—Na1B—Na2B—O2B	117.82 (10)
O2A—Na1A—Na2A—S2A	−75.71 (5)	S2B—Na1B—Na2B—O2B	92.11 (6)
C68A—Na1A—Na2A—S2A	5.54 (5)	O8B—Na2B—O1B—C1B	−165.0 (2)
O5A—Na1A—O1A—C1A	173.17 (19)	O5B—Na2B—O1B—C1B	174.0 (2)
O8A—Na1A—O1A—C1A	169.47 (17)	O3B—Na2B—O1B—C1B	−0.9 (3)
O3A—Na1A—O1A—C1A	2.4 (2)	O4B—Na2B—O1B—C1B	75.31 (19)
O4A—Na1A—O1A—C1A	−77.62 (18)	O2B—Na2B—O1B—C1B	−74.3 (2)
O2A—Na1A—O1A—C1A	67.53 (18)	Na1B—Na2B—O1B—C1B	−173.26 (18)
C68A—Na1A—O1A—C1A	137.0 (3)	O8B—Na2B—O1B—C53B	17.9 (3)
Na2A—Na1A—O1A—C1A	167.59 (17)	O5B—Na2B—O1B—C53B	−3.12 (18)
O5A—Na1A—O1A—C53A	0.63 (15)	O3B—Na2B—O1B—C53B	−177.93 (18)
O8A—Na1A—O1A—C53A	−3.1 (2)	O4B—Na2B—O1B—C53B	−101.76 (18)
O3A—Na1A—O1A—C53A	−170.14 (15)	O2B—Na2B—O1B—C53B	108.63 (18)
O4A—Na1A—O1A—C53A	109.84 (16)	Na1B—Na2B—O1B—C53B	9.67 (19)
O2A—Na1A—O1A—C53A	−105.00 (16)	O8B—Na2B—O2B—C17B	−141.56 (18)
C68A—Na1A—O1A—C53A	−35.6 (3)	O5B—Na2B—O2B—C17B	139.04 (18)
Na2A—Na1A—O1A—C53A	−4.94 (16)	O3B—Na2B—O2B—C17B	−73.38 (19)
O5A—Na1A—O2A—C17A	−133.20 (19)	O1B—Na2B—O2B—C17B	72.35 (18)
O8A—Na1A—O2A—C17A	145.74 (19)	O4B—Na2B—O2B—C17B	1.9 (3)
O3A—Na1A—O2A—C17A	78.66 (19)	Na1B—Na2B—O2B—C17B	179.92 (18)
O1A—Na1A—O2A—C17A	−65.7 (2)	O8B—Na2B—O2B—C45B	39.9 (3)
O4A—Na1A—O2A—C17A	7.9 (3)	O5B—Na2B—O2B—C45B	−39.5 (3)
C68A—Na1A—O2A—C17A	128.3 (2)	O3B—Na2B—O2B—C45B	108.1 (3)
Na2A—Na1A—O2A—C17A	−173.77 (19)	O1B—Na2B—O2B—C45B	−106.1 (3)
O5A—Na1A—O2A—C45A	55.97 (17)	O4B—Na2B—O2B—C45B	−176.6 (3)
O8A—Na1A—O2A—C45A	−25.09 (17)	Na1B—Na2B—O2B—C45B	1.4 (3)
O3A—Na1A—O2A—C45A	−92.16 (16)	O8B—Na2B—O3B—C28B	173.4 (2)
O1A—Na1A—O2A—C45A	123.47 (16)	O5B—Na2B—O3B—C28B	−168.52 (19)
O4A—Na1A—O2A—C45A	−162.95 (16)	O1B—Na2B—O3B—C28B	2.8 (3)
C68A—Na1A—O2A—C45A	−42.50 (16)	O4B—Na2B—O3B—C28B	−72.63 (19)
Na2A—Na1A—O2A—C45A	15.41 (17)	O2B—Na2B—O3B—C28B	76.19 (19)
O5A—Na1A—O3A—C28A	−175.66 (19)	Na1B—Na2B—O3B—C28B	175.24 (17)
O8A—Na1A—O3A—C28A	174.7 (2)	O8B—Na2B—O3B—C67B	−6.52 (17)
O1A—Na1A—O3A—C28A	−12.5 (3)	O5B—Na2B—O3B—C67B	11.5 (3)
O4A—Na1A—O3A—C28A	65.4 (2)	O1B—Na2B—O3B—C67B	−177.13 (17)
O2A—Na1A—O3A—C28A	−80.62 (19)	O4B—Na2B—O3B—C67B	107.43 (17)
C68A—Na1A—O3A—C28A	−178.7 (2)	O2B—Na2B—O3B—C67B	−103.75 (17)
Na2A—Na1A—O3A—C28A	−177.65 (18)	Na1B—Na2B—O3B—C67B	−4.69 (18)
O5A—Na1A—O3A—C67A	7.6 (2)	O8B—Na2B—O4B—C39B	137.27 (17)
O8A—Na1A—O3A—C67A	−2.07 (15)	O5B—Na2B—O4B—C39B	−142.81 (17)
O1A—Na1A—O3A—C67A	170.74 (15)	O3B—Na2B—O4B—C39B	71.46 (17)
O4A—Na1A—O3A—C67A	−111.30 (16)	O1B—Na2B—O4B—C39B	−73.85 (17)
O2A—Na1A—O3A—C67A	102.66 (16)	O2B—Na2B—O4B—C39B	−3.3 (2)
C68A—Na1A—O3A—C67A	4.59 (14)	Na1B—Na2B—O4B—C39B	178.79 (16)
Na2A—Na1A—O3A—C67A	5.62 (16)	O8B—Na2B—O4B—C49B	−58.28 (17)
O5A—Na1A—O4A—C39A	142.67 (17)	O5B—Na2B—O4B—C49B	21.64 (16)
O8A—Na1A—O4A—C39A	−133.95 (17)	O3B—Na2B—O4B—C49B	−124.09 (16)
O3A—Na1A—O4A—C39A	−65.22 (18)	O1B—Na2B—O4B—C49B	90.59 (15)
O1A—Na1A—O4A—C39A	76.65 (18)	O2B—Na2B—O4B—C49B	161.10 (16)
O2A—Na1A—O4A—C39A	2.0 (2)	Na1B—Na2B—O4B—C49B	−16.76 (16)
C68A—Na1A—O4A—C39A	−112.29 (18)	O8B—Na2B—O5B—C54B	−165.4 (3)
Na2A—Na1A—O4A—C39A	−176.21 (16)	O3B—Na2B—O5B—C54B	177.5 (2)
O5A—Na1A—O4A—C49A	−39.0 (2)	O1B—Na2B—O5B—C54B	2.9 (2)
O8A—Na1A—O4A—C49A	44.4 (2)	O4B—Na2B—O5B—C54B	84.5 (2)
O3A—Na1A—O4A—C49A	113.1 (2)	O2B—Na2B—O5B—C54B	−74.0 (2)
O1A—Na1A—O4A—C49A	−105.0 (2)	Na1B—Na2B—O5B—C54B	−158.2 (3)
O2A—Na1A—O4A—C49A	−179.6 (2)	O8B—Na2B—O5B—Na1B	−7.18 (9)
C68A—Na1A—O4A—C49A	66.1 (2)	O3B—Na2B—O5B—Na1B	−24.3 (2)
Na2A—Na1A—O4A—C49A	2.2 (2)	O1B—Na2B—O5B—Na1B	161.05 (10)
O8A—Na1A—O5A—C54A	175.6 (2)	O4B—Na2B—O5B—Na1B	−117.32 (8)
O3A—Na1A—O5A—C54A	166.31 (17)	O2B—Na2B—O5B—Na1B	84.14 (9)
O1A—Na1A—O5A—C54A	−2.44 (18)	O6B—Na1B—O5B—C54B	−21.3 (3)
O4A—Na1A—O5A—C54A	−78.8 (2)	O8B—Na1B—O5B—C54B	161.4 (3)
O2A—Na1A—O5A—C54A	78.6 (2)	O2W—Na1B—O5B—C54B	62.5 (3)
C68A—Na1A—O5A—C54A	168.67 (19)	O10B—Na1B—O5B—C54B	−143.3 (3)
Na2A—Na1A—O5A—C54A	169.3 (2)	S2B—Na1B—O5B—C54B	−179.1 (2)
O8A—Na1A—O5A—Na2A	6.32 (7)	Na2B—Na1B—O5B—C54B	154.1 (3)
O3A—Na1A—O5A—Na2A	−2.95 (18)	O6B—Na1B—O5B—Na2B	−175.43 (12)
O1A—Na1A—O5A—Na2A	−171.71 (9)	O8B—Na1B—O5B—Na2B	7.23 (9)
O4A—Na1A—O5A—Na2A	111.95 (8)	O2W—Na1B—O5B—Na2B	−91.66 (8)
O2A—Na1A—O5A—Na2A	−90.70 (8)	O10B—Na1B—O5B—Na2B	62.57 (14)
C68A—Na1A—O5A—Na2A	−0.59 (9)	S2B—Na1B—O5B—Na2B	26.81 (14)
O1W—Na2A—O5A—C54A	92.4 (3)	O7B—S1B—O6B—Na1B	165.1 (2)
O8A—Na2A—O5A—C54A	−173.7 (2)	N1B—S1B—O6B—Na1B	40.9 (3)
O7A—Na2A—O5A—C54A	−15.4 (2)	C55B—S1B—O6B—Na1B	−72.7 (2)
O10A—Na2A—O5A—C54A	−124.5 (2)	O8B—Na1B—O6B—S1B	−8.6 (4)
S1A—Na2A—O5A—C54A	5.5 (2)	O5B—Na1B—O6B—S1B	−14.3 (2)
S2A—Na2A—O5A—C54A	−158.54 (19)	O2W—Na1B—O6B—S1B	−136.5 (2)
Na1A—Na2A—O5A—C54A	−167.4 (3)	O10B—Na1B—O6B—S1B	116.1 (2)
O1W—Na2A—O5A—Na1A	−100.18 (13)	S2B—Na1B—O6B—S1B	139.88 (16)
O8A—Na2A—O5A—Na1A	−6.32 (7)	Na2B—Na1B—O6B—S1B	−17.5 (3)
O7A—Na2A—O5A—Na1A	151.99 (10)	O6B—Na1B—O8B—C68B	155.6 (3)
O10A—Na2A—O5A—Na1A	42.92 (15)	O5B—Na1B—O8B—C68B	161.3 (3)
S1A—Na2A—O5A—Na1A	172.96 (9)	O2W—Na1B—O8B—C68B	−78.8 (3)
S2A—Na2A—O5A—Na1A	8.88 (12)	O10B—Na1B—O8B—C68B	21.2 (3)
O6A—S1A—O7A—Na2A	59.17 (15)	S2B—Na1B—O8B—C68B	−3.4 (3)
N1A—S1A—O7A—Na2A	−70.82 (16)	Na2B—Na1B—O8B—C68B	168.6 (4)
C55A—S1A—O7A—Na2A	178.19 (11)	O6B—Na1B—O8B—Na2B	−13.0 (3)
O1W—Na2A—O7A—S1A	−71.90 (13)	O5B—Na1B—O8B—Na2B	−7.32 (9)
O8A—Na2A—O7A—S1A	89.69 (18)	O2W—Na1B—O8B—Na2B	112.60 (9)
O5A—Na2A—O7A—S1A	51.12 (12)	O10B—Na1B—O8B—Na2B	−147.39 (13)
O10A—Na2A—O7A—S1A	−168.69 (11)	S2B—Na1B—O8B—Na2B	−171.98 (12)
S2A—Na2A—O7A—S1A	−166.62 (7)	O5B—Na2B—O8B—C68B	−163.0 (3)
Na1A—Na2A—O7A—S1A	69.01 (13)	O3B—Na2B—O8B—C68B	6.7 (2)
O5A—Na1A—O8A—C68A	−160.23 (18)	O1B—Na2B—O8B—C68B	176.8 (2)
O3A—Na1A—O8A—C68A	14.71 (16)	O4B—Na2B—O8B—C68B	−70.9 (3)
O1A—Na1A—O8A—C68A	−156.71 (17)	O2B—Na2B—O8B—C68B	89.2 (2)
O4A—Na1A—O8A—C68A	97.07 (17)	Na1B—Na2B—O8B—C68B	−170.5 (3)
O2A—Na1A—O8A—C68A	−58.32 (18)	O5B—Na2B—O8B—Na1B	7.47 (10)
Na2A—Na1A—O8A—C68A	−153.9 (2)	O3B—Na2B—O8B—Na1B	177.21 (12)
O5A—Na1A—O8A—Na2A	−6.30 (7)	O1B—Na2B—O8B—Na1B	−12.7 (2)
O3A—Na1A—O8A—Na2A	168.64 (8)	O4B—Na2B—O8B—Na1B	99.64 (9)
O1A—Na1A—O8A—Na2A	−2.78 (17)	O2B—Na2B—O8B—Na1B	−100.25 (9)
O4A—Na1A—O8A—Na2A	−109.00 (7)	O9B—S2B—O10B—Na1B	−51.25 (17)
O2A—Na1A—O8A—Na2A	95.61 (7)	N3B—S2B—O10B—Na1B	79.23 (17)
C68A—Na1A—O8A—Na2A	153.9 (2)	C69B—S2B—O10B—Na1B	−170.60 (12)
O1W—Na2A—O8A—C68A	−81.3 (2)	O6B—Na1B—O10B—S2B	146.46 (12)
O7A—Na2A—O8A—C68A	115.8 (2)	O8B—Na1B—O10B—S2B	−56.26 (13)
O5A—Na2A—O8A—C68A	153.7 (2)	O5B—Na1B—O10B—S2B	−112.80 (17)
O10A—Na2A—O8A—C68A	2.4 (2)	O2W—Na1B—O10B—S2B	44.87 (15)
S1A—Na2A—O8A—C68A	152.73 (18)	Na2B—Na1B—O10B—S2B	−76.87 (15)
S2A—Na2A—O8A—C68A	−14.15 (19)	O7B—S1B—N1B—C54B	−168.6 (3)
Na1A—Na2A—O8A—C68A	147.5 (2)	O6B—S1B—N1B—C54B	−39.9 (3)
O1W—Na2A—O8A—Na1A	131.15 (6)	C55B—S1B—N1B—C54B	75.9 (3)
O7A—Na2A—O8A—Na1A	−31.73 (16)	O10B—S2B—N3B—C68B	−46.2 (3)
O5A—Na2A—O8A—Na1A	6.15 (7)	O9B—S2B—N3B—C68B	84.8 (3)
O10A—Na2A—O8A—Na1A	−145.11 (9)	C69B—S2B—N3B—C68B	−161.2 (3)
S1A—Na2A—O8A—Na1A	5.20 (11)	Na1B—S2B—N3B—C68B	3.3 (3)
S2A—Na2A—O8A—Na1A	−161.68 (8)	C53B—O1B—C1B—C2B	88.6 (2)
O9A—S2A—O10A—Na2A	−63.10 (15)	Na2B—O1B—C1B—C2B	−88.6 (2)
N3A—S2A—O10A—Na2A	66.53 (16)	C53B—O1B—C1B—C6B	−92.8 (3)
C69A—S2A—O10A—Na2A	177.43 (11)	Na2B—O1B—C1B—C6B	90.0 (2)
O1W—Na2A—O10A—S2A	62.22 (15)	C6B—C1B—C2B—C3B	3.4 (3)
O8A—Na2A—O10A—S2A	−39.27 (12)	O1B—C1B—C2B—C3B	−178.09 (18)
O7A—Na2A—O10A—S2A	176.36 (12)	C6B—C1B—C2B—C44B	−174.0 (2)
O5A—Na2A—O10A—S2A	−88.70 (18)	O1B—C1B—C2B—C44B	4.6 (3)
S1A—Na2A—O10A—S2A	169.74 (7)	C1B—C2B—C3B—C4B	−0.5 (3)
Na1A—Na2A—O10A—S2A	−62.04 (14)	C44B—C2B—C3B—C4B	176.9 (2)
O6A—S1A—N1A—C54A	−95.3 (2)	C2B—C3B—C4B—C5B	−1.6 (3)
O7A—S1A—N1A—C54A	36.3 (3)	C2B—C3B—C4B—C7B	177.1 (2)
C55A—S1A—N1A—C54A	150.5 (2)	C3B—C4B—C5B—C6B	0.9 (3)
Na2A—S1A—N1A—C54A	−1.8 (2)	C7B—C4B—C5B—C6B	−177.8 (2)
O9A—S2A—N3A—C68A	82.5 (2)	C2B—C1B—C6B—C5B	−4.0 (3)
O10A—S2A—N3A—C68A	−49.1 (2)	O1B—C1B—C6B—C5B	177.46 (18)
C69A—S2A—N3A—C68A	−164.5 (2)	C2B—C1B—C6B—C11B	171.3 (2)
Na2A—S2A—N3A—C68A	−12.8 (2)	O1B—C1B—C6B—C11B	−7.3 (3)
C53A—O1A—C1A—C6A	86.3 (2)	C4B—C5B—C6B—C1B	1.8 (3)
Na1A—O1A—C1A—C6A	−86.7 (2)	C4B—C5B—C6B—C11B	−173.5 (2)
C53A—O1A—C1A—C2A	−94.9 (2)	C5B—C4B—C7B—C9B	−168.8 (3)
Na1A—O1A—C1A—C2A	92.1 (2)	C3B—C4B—C7B—C9B	12.6 (4)
C6A—C1A—C2A—C3A	−4.4 (3)	C5B—C4B—C7B—C10B	72.8 (3)
O1A—C1A—C2A—C3A	176.82 (19)	C3B—C4B—C7B—C10B	−105.8 (3)
C6A—C1A—C2A—C44A	171.0 (2)	C5B—C4B—C7B—C8B	−46.8 (3)
O1A—C1A—C2A—C44A	−7.8 (3)	C3B—C4B—C7B—C8B	134.6 (3)
C1A—C2A—C3A—C4A	2.3 (3)	C1B—C6B—C11B—C12B	−96.5 (3)
C44A—C2A—C3A—C4A	−173.2 (2)	C5B—C6B—C11B—C12B	78.7 (3)
C2A—C3A—C4A—C5A	0.5 (4)	C6B—C11B—C12B—C17B	96.9 (3)
C2A—C3A—C4A—C7A	179.0 (2)	C6B—C11B—C12B—C13B	−78.8 (3)
C3A—C4A—C5A—C6A	−1.4 (4)	C17B—C12B—C13B—C14B	−1.5 (4)
C7A—C4A—C5A—C6A	−179.8 (2)	C11B—C12B—C13B—C14B	174.3 (2)
O1A—C1A—C6A—C5A	−177.65 (19)	C12B—C13B—C14B—C15B	−2.9 (4)
C2A—C1A—C6A—C5A	3.6 (3)	C12B—C13B—C14B—C18B	178.0 (2)
O1A—C1A—C6A—C11A	4.8 (3)	C13B—C14B—C15B—C16B	2.8 (4)
C2A—C1A—C6A—C11A	−174.0 (2)	C18B—C14B—C15B—C16B	−178.0 (2)
C4A—C5A—C6A—C1A	−0.6 (3)	C14B—C15B—C16B—C17B	1.7 (4)
C4A—C5A—C6A—C11A	177.0 (2)	C14B—C15B—C16B—C22B	−175.1 (2)
C5A—C4A—C7A—C9A	114.1 (4)	C45B—O2B—C17B—C12B	88.2 (4)
C3A—C4A—C7A—C9A	−64.3 (4)	Na2B—O2B—C17B—C12B	−90.4 (3)
C5A—C4A—C7A—C10A	−6.1 (4)	C45B—O2B—C17B—C16B	−92.1 (3)
C3A—C4A—C7A—C10A	175.5 (3)	Na2B—O2B—C17B—C16B	89.2 (3)
C5A—C4A—C7A—C8A	−127.7 (3)	C13B—C12B—C17B—O2B	−174.2 (2)
C3A—C4A—C7A—C8A	53.9 (4)	C11B—C12B—C17B—O2B	10.1 (4)
C1A—C6A—C11A—C12A	103.8 (2)	C13B—C12B—C17B—C16B	6.2 (4)
C5A—C6A—C11A—C12A	−73.8 (3)	C11B—C12B—C17B—C16B	−169.5 (2)
C6A—C11A—C12A—C13A	78.2 (3)	C15B—C16B—C17B—O2B	174.1 (2)
C6A—C11A—C12A—C17A	−94.6 (3)	C22B—C16B—C17B—O2B	−9.3 (4)
C17A—C12A—C13A—C14A	4.3 (4)	C15B—C16B—C17B—C12B	−6.3 (4)
C11A—C12A—C13A—C14A	−168.8 (2)	C22B—C16B—C17B—C12B	170.3 (2)
C12A—C13A—C14A—C15A	0.1 (4)	C13B—C14B—C18B—C20B	117.4 (3)
C12A—C13A—C14A—C18A	−178.8 (3)	C15B—C14B—C18B—C20B	−61.7 (4)
C13A—C14A—C15A—C16A	−2.6 (4)	C13B—C14B—C18B—C21B	−4.2 (4)
C18A—C14A—C15A—C16A	176.2 (3)	C15B—C14B—C18B—C21B	176.6 (3)
C14A—C15A—C16A—C17A	0.6 (4)	C13B—C14B—C18B—C19B	−123.1 (3)
C14A—C15A—C16A—C22A	178.1 (3)	C15B—C14B—C18B—C19B	57.8 (3)
C45A—O2A—C17A—C16A	78.1 (3)	C15B—C16B—C22B—C23B	79.4 (3)
Na1A—O2A—C17A—C16A	−92.7 (3)	C17B—C16B—C22B—C23B	−97.2 (3)
C45A—O2A—C17A—C12A	−103.7 (3)	C16B—C22B—C23B—C28B	97.0 (3)
Na1A—O2A—C17A—C12A	85.6 (3)	C16B—C22B—C23B—C24B	−79.3 (3)
C15A—C16A—C17A—O2A	−177.8 (2)	C28B—C23B—C24B—C25B	−1.3 (3)
C22A—C16A—C17A—O2A	4.7 (4)	C22B—C23B—C24B—C25B	175.0 (2)
C15A—C16A—C17A—C12A	3.9 (4)	C23B—C24B—C25B—C26B	−2.0 (3)
C22A—C16A—C17A—C12A	−173.6 (2)	C23B—C24B—C25B—C29B	178.3 (2)
C13A—C12A—C17A—O2A	175.5 (2)	C24B—C25B—C26B—C27B	2.2 (3)
C11A—C12A—C17A—O2A	−11.7 (4)	C29B—C25B—C26B—C27B	−178.1 (2)
C13A—C12A—C17A—C16A	−6.3 (4)	C25B—C26B—C27B—C28B	0.8 (3)
C11A—C12A—C17A—C16A	166.6 (2)	C25B—C26B—C27B—C33B	−176.8 (2)
C15A—C14A—C18A—C19A	−107.1 (3)	C24B—C23B—C28B—O3B	−175.95 (19)
C13A—C14A—C18A—C19A	71.6 (3)	C22B—C23B—C28B—O3B	7.7 (3)
C15A—C14A—C18A—C20A	14.6 (4)	C24B—C23B—C28B—C27B	4.6 (3)
C13A—C14A—C18A—C20A	−166.6 (3)	C22B—C23B—C28B—C27B	−171.7 (2)
C15A—C14A—C18A—C21A	134.4 (3)	C67B—O3B—C28B—C23B	88.3 (3)
C13A—C14A—C18A—C21A	−46.9 (4)	Na2B—O3B—C28B—C23B	−91.7 (2)
C15A—C16A—C22A—C23A	−82.6 (3)	C67B—O3B—C28B—C27B	−92.3 (2)
C17A—C16A—C22A—C23A	94.9 (3)	Na2B—O3B—C28B—C27B	87.8 (2)
C16A—C22A—C23A—C24A	85.2 (3)	C26B—C27B—C28B—C23B	−4.4 (3)
C16A—C22A—C23A—C28A	−94.4 (3)	C33B—C27B—C28B—C23B	173.2 (2)
C28A—C23A—C24A—C25A	−0.5 (4)	C26B—C27B—C28B—O3B	176.22 (19)
C22A—C23A—C24A—C25A	179.9 (2)	C33B—C27B—C28B—O3B	−6.2 (3)
C23A—C24A—C25A—C26A	3.1 (4)	C24B—C25B—C29B—C32B	112.9 (3)
C23A—C24A—C25A—C29A	−175.4 (3)	C26B—C25B—C29B—C32B	−66.8 (3)
C24A—C25A—C26A—C27A	−1.4 (4)	C24B—C25B—C29B—C31B	−6.9 (4)
C29A—C25A—C26A—C27A	177.2 (2)	C26B—C25B—C29B—C31B	173.4 (2)
C25A—C26A—C27A—C28A	−2.8 (4)	C24B—C25B—C29B—C30B	−124.3 (3)
C25A—C26A—C27A—C33A	172.1 (2)	C26B—C25B—C29B—C30B	56.1 (3)
C26A—C27A—C28A—O3A	−173.5 (2)	C28B—C27B—C33B—C34B	−99.4 (2)
C33A—C27A—C28A—O3A	11.8 (3)	C26B—C27B—C33B—C34B	78.1 (3)
C26A—C27A—C28A—C23A	5.4 (3)	C27B—C33B—C34B—C35B	−78.1 (3)
C33A—C27A—C28A—C23A	−169.3 (2)	C27B—C33B—C34B—C39B	97.2 (3)
C67A—O3A—C28A—C27A	91.7 (2)	C39B—C34B—C35B—C36B	−1.6 (4)
Na1A—O3A—C28A—C27A	−85.2 (2)	C33B—C34B—C35B—C36B	174.0 (2)
C67A—O3A—C28A—C23A	−87.2 (2)	C34B—C35B—C36B—C37B	−2.6 (4)
Na1A—O3A—C28A—C23A	95.9 (2)	C34B—C35B—C36B—C40B	175.9 (2)
C24A—C23A—C28A—C27A	−3.9 (3)	C35B—C36B—C37B—C38B	2.1 (4)
C22A—C23A—C28A—C27A	175.7 (2)	C40B—C36B—C37B—C38B	−176.4 (2)
C24A—C23A—C28A—O3A	175.0 (2)	C36B—C37B—C38B—C39B	2.6 (4)
C22A—C23A—C28A—O3A	−5.4 (3)	C36B—C37B—C38B—C44B	−173.5 (2)
C24A—C25A—C29A—C31A	106.7 (4)	C49B—O4B—C39B—C34B	106.8 (2)
C26A—C25A—C29A—C31A	−71.7 (4)	Na2B—O4B—C39B—C34B	−88.2 (2)
C24A—C25A—C29A—C30A	−12.8 (5)	C49B—O4B—C39B—C38B	−74.6 (3)
C26A—C25A—C29A—C30A	168.7 (3)	Na2B—O4B—C39B—C38B	90.3 (2)
C24A—C25A—C29A—C32A	−133.6 (4)	C35B—C34B—C39B—O4B	−175.1 (2)
C26A—C25A—C29A—C32A	47.9 (4)	C33B—C34B—C39B—O4B	9.5 (3)
C28A—C27A—C33A—C34A	94.2 (3)	C35B—C34B—C39B—C38B	6.4 (4)
C26A—C27A—C33A—C34A	−80.4 (3)	C33B—C34B—C39B—C38B	−169.0 (2)
C27A—C33A—C34A—C39A	−101.3 (3)	C37B—C38B—C39B—O4B	174.6 (2)
C27A—C33A—C34A—C35A	74.0 (3)	C44B—C38B—C39B—O4B	−9.4 (3)
C39A—C34A—C35A—C36A	2.2 (4)	C37B—C38B—C39B—C34B	−6.9 (3)
C33A—C34A—C35A—C36A	−173.3 (2)	C44B—C38B—C39B—C34B	169.1 (2)
C34A—C35A—C36A—C37A	1.9 (4)	C37B—C36B—C40B—C43B	−128.4 (3)
C34A—C35A—C36A—C40A	179.7 (3)	C35B—C36B—C40B—C43B	53.2 (4)
C35A—C36A—C37A—C38A	−2.9 (4)	C37B—C36B—C40B—C41B	−7.4 (4)
C40A—C36A—C37A—C38A	179.3 (3)	C35B—C36B—C40B—C41B	174.2 (3)
C36A—C37A—C38A—C39A	−0.4 (4)	C37B—C36B—C40B—C42B	111.3 (3)
C36A—C37A—C38A—C44A	175.8 (2)	C35B—C36B—C40B—C42B	−67.1 (3)
C35A—C34A—C39A—O4A	174.0 (2)	C1B—C2B—C44B—C38B	98.4 (2)
C33A—C34A—C39A—O4A	−10.6 (4)	C3B—C2B—C44B—C38B	−78.9 (3)
C35A—C34A—C39A—C38A	−5.6 (4)	C37B—C38B—C44B—C2B	80.0 (3)
C33A—C34A—C39A—C38A	169.7 (2)	C39B—C38B—C44B—C2B	−96.0 (3)
C49A—O4A—C39A—C34A	−91.3 (3)	C17B—O2B—C45B—C46B	−174.0 (5)
Na1A—O4A—C39A—C34A	87.3 (3)	Na2B—O2B—C45B—C46B	4.7 (7)
C49A—O4A—C39A—C38A	88.3 (3)	O2B—C45B—C46B—C47B	177.6 (5)
Na1A—O4A—C39A—C38A	−93.0 (2)	C45B—C46B—C47B—C48B	14.3 (11)
C37A—C38A—C39A—C34A	4.8 (4)	C39B—O4B—C49B—C50B	−78.6 (3)
C44A—C38A—C39A—C34A	−171.3 (2)	Na2B—O4B—C49B—C50B	114.8 (2)
C37A—C38A—C39A—O4A	−174.9 (2)	O4B—C49B—C50B—C51B	−77.2 (3)
C44A—C38A—C39A—O4A	9.1 (3)	C49B—C50B—C51B—C52B	−173.4 (4)
C37A—C36A—C40A—C41A	64.1 (4)	C1B—O1B—C53B—C54B	−174.4 (2)
C35A—C36A—C40A—C41A	−113.6 (3)	Na2B—O1B—C53B—C54B	3.1 (3)
C37A—C36A—C40A—C42A	−175.8 (3)	Na2B—O5B—C54B—N1B	176.0 (3)
C35A—C36A—C40A—C42A	6.4 (4)	Na1B—O5B—C54B—N1B	26.1 (5)
C37A—C36A—C40A—C43A	−54.4 (4)	Na2B—O5B—C54B—C53B	−2.2 (4)
C35A—C36A—C40A—C43A	127.9 (3)	Na1B—O5B—C54B—C53B	−152.1 (2)
C3A—C2A—C44A—C38A	79.7 (3)	S1B—N1B—C54B—O5B	8.4 (5)
C1A—C2A—C44A—C38A	−95.6 (2)	S1B—N1B—C54B—C53B	−173.3 (2)
C37A—C38A—C44A—C2A	−78.6 (3)	O1B—C53B—C54B—O5B	−0.7 (4)
C39A—C38A—C44A—C2A	97.3 (3)	O1B—C53B—C54B—N1B	−179.2 (2)
C17A—O2A—C45A—C46A	100.6 (3)	O7B—S1B—C55B—C56B	140.4 (3)
Na1A—O2A—C45A—C46A	−86.8 (3)	O6B—S1B—C55B—C56B	14.9 (3)
O2A—C45A—C46A—C47A	−179.0 (3)	N1B—S1B—C55B—C56B	−106.3 (3)
C45A—C46A—C47A—C48A	−174.6 (4)	O7B—S1B—C55B—C64B	−44.2 (3)
C39A—O4A—C49A—C50A	−163.0 (3)	O6B—S1B—C55B—C64B	−169.8 (2)
Na1A—O4A—C49A—C50A	18.4 (4)	N1B—S1B—C55B—C64B	69.0 (3)
O4A—C49A—C50A—C51A	174.3 (3)	C64B—C55B—C56B—C57B	−5.1 (6)
C49A—C50A—C51A—C52A	178.4 (4)	S1B—C55B—C56B—C57B	170.2 (3)
C1A—O1A—C53A—C54A	−173.02 (18)	C55B—C56B—C57B—C58B	2.5 (7)
Na1A—O1A—C53A—C54A	0.7 (2)	C56B—C57B—C58B—C59B	3.8 (7)
Na1A—O5A—C54A—N1A	−175.4 (2)	C57B—C58B—C59B—C60B	175.1 (4)
Na2A—O5A—C54A—N1A	−10.1 (4)	C57B—C58B—C59B—C64B	−7.8 (6)
Na1A—O5A—C54A—C53A	3.8 (3)	C58B—C59B—C60B—C61B	175.4 (4)
Na2A—O5A—C54A—C53A	169.13 (16)	C64B—C59B—C60B—C61B	−1.7 (5)
S1A—N1A—C54A—O5A	6.9 (4)	C58B—C59B—C60B—N2B	−1.4 (6)
S1A—N1A—C54A—C53A	−172.35 (17)	C64B—C59B—C60B—N2B	−178.5 (3)
O1A—C53A—C54A—O5A	−2.8 (3)	C65B—N2B—C60B—C61B	110.1 (6)
O1A—C53A—C54A—N1A	176.5 (2)	C66B—N2B—C60B—C61B	−19.5 (6)
O6A—S1A—C55A—C56A	10.1 (2)	C65B—N2B—C60B—C59B	−73.1 (6)
O7A—S1A—C55A—C56A	−113.5 (2)	C66B—N2B—C60B—C59B	157.2 (4)
N1A—S1A—C55A—C56A	127.3 (2)	C59B—C60B—C61B—C62B	−1.1 (6)
Na2A—S1A—C55A—C56A	−111.4 (2)	N2B—C60B—C61B—C62B	175.7 (4)
O6A—S1A—C55A—C64A	−173.9 (2)	C60B—C61B—C62B—C63B	2.8 (6)
O7A—S1A—C55A—C64A	62.5 (2)	C61B—C62B—C63B—C64B	−1.5 (5)
N1A—S1A—C55A—C64A	−56.7 (2)	C62B—C63B—C64B—C55B	179.4 (3)
Na2A—S1A—C55A—C64A	64.6 (3)	C62B—C63B—C64B—C59B	−1.3 (5)
C64A—C55A—C56A—C57A	−2.5 (4)	C56B—C55B—C64B—C63B	−179.6 (3)
S1A—C55A—C56A—C57A	173.5 (2)	S1B—C55B—C64B—C63B	5.3 (4)
C55A—C56A—C57A—C58A	2.6 (4)	C56B—C55B—C64B—C59B	1.2 (5)
C56A—C57A—C58A—C59A	1.7 (4)	S1B—C55B—C64B—C59B	−173.9 (2)
C57A—C58A—C59A—C64A	−5.9 (4)	C60B—C59B—C64B—C63B	2.9 (5)
C57A—C58A—C59A—C60A	178.3 (2)	C58B—C59B—C64B—C63B	−174.2 (3)
C66A—N2A—C60A—C61A	−17.4 (4)	C60B—C59B—C64B—C55B	−177.8 (3)
C65A—N2A—C60A—C61A	118.5 (3)	C58B—C59B—C64B—C55B	5.1 (5)
C66A—N2A—C60A—C59A	158.3 (3)	C28B—O3B—C67B—C68B	−174.0 (2)
C65A—N2A—C60A—C59A	−65.9 (4)	Na2B—O3B—C67B—C68B	5.9 (3)
C58A—C59A—C60A—C61A	172.4 (3)	Na1B—O8B—C68B—N3B	7.7 (5)
C64A—C59A—C60A—C61A	−3.4 (4)	Na2B—O8B—C68B—N3B	174.6 (3)
C58A—C59A—C60A—N2A	−3.4 (4)	Na1B—O8B—C68B—C67B	−173.0 (2)
C64A—C59A—C60A—N2A	−179.2 (2)	Na2B—O8B—C68B—C67B	−6.1 (4)
N2A—C60A—C61A—C62A	177.1 (3)	S2B—N3B—C68B—O8B	−7.0 (5)
C59A—C60A—C61A—C62A	1.5 (4)	S2B—N3B—C68B—C67B	173.6 (2)
C60A—C61A—C62A—C63A	0.7 (5)	O3B—C67B—C68B—O8B	0.0 (4)
C61A—C62A—C63A—C64A	−0.9 (4)	O3B—C67B—C68B—N3B	179.4 (2)
C62A—C63A—C64A—C55A	−179.5 (2)	O10B—S2B—C69B—C70B	114.4 (3)
C62A—C63A—C64A—C59A	−1.1 (4)	O9B—S2B—C69B—C70B	−8.9 (3)
C56A—C55A—C64A—C63A	176.7 (2)	N3B—S2B—C69B—C70B	−126.8 (3)
S1A—C55A—C64A—C63A	0.9 (3)	Na1B—S2B—C69B—C70B	95.8 (4)
C56A—C55A—C64A—C59A	−1.6 (3)	O10B—S2B—C69B—C78B	−67.6 (3)
S1A—C55A—C64A—C59A	−177.43 (17)	O9B—S2B—C69B—C78B	169.1 (3)
C58A—C59A—C64A—C63A	−172.7 (2)	N3B—S2B—C69B—C78B	51.2 (3)
C60A—C59A—C64A—C63A	3.2 (3)	Na1B—S2B—C69B—C78B	−86.3 (3)
C58A—C59A—C64A—C55A	5.7 (3)	C78B—C69B—C70B—C71B	−1.3 (6)
C60A—C59A—C64A—C55A	−178.3 (2)	S2B—C69B—C70B—C71B	176.7 (3)
C28A—O3A—C67A—C68A	174.81 (19)	C69B—C70B—C71B—C72B	0.0 (6)
Na1A—O3A—C67A—C68A	−7.8 (2)	C70B—C71B—C72B—C73B	0.2 (6)
Na1A—O8A—C68A—N3A	155.8 (2)	C71B—C72B—C73B—C74B	−176.8 (3)
Na2A—O8A—C68A—N3A	12.6 (4)	C71B—C72B—C73B—C78B	0.9 (5)
Na1A—O8A—C68A—C67A	−25.2 (3)	C72B—C73B—C74B—C75B	176.5 (3)
Na2A—O8A—C68A—C67A	−168.43 (16)	C78B—C73B—C74B—C75B	−1.2 (5)
Na2A—O8A—C68A—Na1A	−143.2 (3)	C72B—C73B—C74B—N4B	−1.1 (5)
S2A—N3A—C68A—O8A	6.5 (4)	C78B—C73B—C74B—N4B	−178.7 (3)
S2A—N3A—C68A—C67A	−172.55 (17)	C80B—N4B—C74B—C75B	−35.1 (5)
S2A—N3A—C68A—Na1A	61.7 (6)	C79B—N4B—C74B—C75B	94.4 (4)
O3A—C67A—C68A—O8A	21.7 (3)	C80B—N4B—C74B—C73B	142.4 (3)
O3A—C67A—C68A—N3A	−159.1 (2)	C79B—N4B—C74B—C73B	−88.1 (4)
O3A—C67A—C68A—Na1A	5.19 (16)	C73B—C74B—C75B—C76B	3.1 (6)
O5A—Na1A—C68A—O8A	19.65 (18)	N4B—C74B—C75B—C76B	−179.4 (4)
O3A—Na1A—C68A—O8A	−161.9 (2)	C74B—C75B—C76B—C77B	−1.5 (6)
O1A—Na1A—C68A—O8A	53.5 (3)	C75B—C76B—C77B—C78B	−2.1 (6)
O4A—Na1A—C68A—O8A	−89.84 (18)	C76B—C77B—C78B—C69B	−173.9 (4)
O2A—Na1A—C68A—O8A	122.81 (18)	C76B—C77B—C78B—C73B	3.9 (5)
Na2A—Na1A—C68A—O8A	19.21 (15)	C70B—C69B—C78B—C77B	−179.9 (4)
O5A—Na1A—C68A—N3A	−52.7 (5)	S2B—C69B—C78B—C77B	2.3 (5)
O8A—Na1A—C68A—N3A	−72.3 (5)	C70B—C69B—C78B—C73B	2.3 (5)
O3A—Na1A—C68A—N3A	125.7 (5)	S2B—C69B—C78B—C73B	−175.6 (2)
O1A—Na1A—C68A—N3A	−18.8 (7)	C72B—C73B—C78B—C77B	180.0 (3)
O4A—Na1A—C68A—N3A	−162.2 (5)	C74B—C73B—C78B—C77B	−2.3 (5)
O2A—Na1A—C68A—N3A	50.5 (5)	C72B—C73B—C78B—C69B	−2.0 (4)
Na2A—Na1A—C68A—N3A	−53.1 (5)	C74B—C73B—C78B—C69B	175.7 (3)
